# Super-resolution imaging and tracking of protein–protein interactions in sub-diffraction cellular space

**DOI:** 10.1038/ncomms5443

**Published:** 2014-07-17

**Authors:** Zhen Liu, Dong Xing, Qian Peter Su, Yun Zhu, Jiamei Zhang, Xinyu Kong, Boxin Xue, Sheng Wang, Hao Sun, Yile Tao, Yujie Sun

**Affiliations:** 1 State Key Laboratory of Biomembrane and Membrane Biotechnology, Biodynamic Optical Imaging Center (BIOPIC), School of Life Sciences, Peking University, Beijing 100871, China; 2 Department of Chemistry and Chemical Biology, Harvard University, Cambridge, Massachusetts 02138, USA; 3 Department of Biochemistry, School of Molecular and Cellular Biology, University of Illinois at Urbana-Champaign, Urbana, Illinois 61801, USA; 4 Biology and Biotechnology, Life Sciences and Bioengineering Center, Worcester Polytechnic Institute, 100 Institute Road, Worcester, Massachusetts 02280, USA

## Abstract

Imaging the location and dynamics of individual interacting protein pairs is essential but often difficult because of the fluorescent background from other paired and non-paired molecules, particularly in the sub-diffraction cellular space. Here we develop a new method combining bimolecular fluorescence complementation and photoactivated localization microscopy for super-resolution imaging and single-molecule tracking of specific protein–protein interactions. The method is used to study the interaction of two abundant proteins, MreB and EF-Tu, in *Escherichia coli* cells. The super-resolution imaging shows interesting distribution and domain sizes of interacting MreB–EF-Tu pairs as a subpopulation of total EF-Tu. The single-molecule tracking of MreB, EF-Tu and MreB–EF-Tu pairs reveals intriguing localization-dependent heterogonous dynamics and provides valuable insights to understanding the roles of MreB–EF-Tu interactions.

## Abstract (web summary)

Protein–protein interactions are ubiquitous in cells and these contacts are crucial for a wide number of cellular processes. Here, the authors present a technique for the super-resolution imaging and tracking of protein–protein interactions in cells.

## Graphical abstract

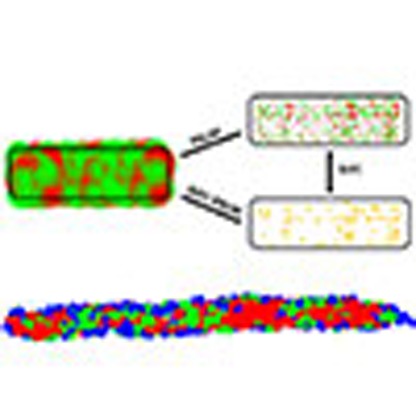

Protein–protein interaction (PPI) is the foundation for most cellular processes. Mass spectrum and biochemistry approaches have identified numerous PPI networks^1^. Meanwhile, accumulating evidence has shown that the functions of PPIs are tightly related to their spatial distribution and temporal dynamics, and therefore direct visualization of PPIs in living cells and organisms is crucial^2^. For a given target protein, imaging its individual PPIs can be very challenging because of several inter-dependent issues, including multiple kinds of interacting partners, high molecule density and heterogonous dynamics, all imaged in a sub-diffraction cellular space.

A typical example is the prokaryotic cell, which, although lacking internal membrane systems, is recently discovered to have subcellular domains and higher-order organization^2^. All of the current imaging approaches have limitations for studying PPIs in such small and crowded systems. For instance, electron microscopy is unsuitable for dynamic imaging of a particular PPI subpopulation because of its poor specificity and low temporal resolution, albeit its exceeding spatial resolution. Fluorescent imaging techniques have high specificity, but optical diffraction disqualifies conventional fluorescence microscopy for imaging the subcellular distribution and dynamics of high-density molecules. Recently developed super-resolution optical imaging techniques, such as stochastic optical reconstruction microscopy (STORM)^3^ and (fluorescent) photoactivated localization microscopy (FPALM/PALM)^4^,^5^, have redefined the resolution barrier and allowed cellular ultrastructures being resolved at ~7 nm resolution^6^. Regarding PPI imaging, two-colour co-localization can be in principle used to identify particular PPIs, but often suffers high background from non-interacting proteins (Fig. 1a) and uncertainty on seemingly overlapped pairs even in super-resolution images due to finite spatial resolution (Fig. 1b). Förster resonance energy transfer (FRET) is a powerful fluorescent approach for studying PPIs, particularly their dynamic processes^7^. However, because of spectral bleed-through and limited energy transfer efficiency, imaging individual FRET pairs in live cells is difficult. It is thus challenging to implement FRET on single-molecule localization-based super-resolution imaging methods such as STORM and FPALM/PALM.

Protein-fragment complementation assay is a special tool set for analysing PPIs, based on enzymatic or fluorescent complementation reporters^8^. Among different protein-fragment complementation assays, bimolecular fluorescence complementation (BiFC) is unique because it does not require any substrates or cofactors, but rather relies solely on complementation of a fluorescent protein. Generally, a fluorescent protein is split and the two complementary yet non-fluorescent fragments are fused to two interacting proteins, respectively. As the complementation of fluorescent protein occurs only when a pair of target PPI brings the two complementary fragments together, BiFC has high specificity and sensitivity for PPI imaging. These advantages have promoted a wide range of applications for BiFC since its invention nearly a decade ago^9^,^10^,^11^. Compared with FRET, BiFC is capable of imaging and tracking single PPIs in live cells because it has higher fluorescence signal (equivalent to the intact fluorescent protein) and much lower background. Given the aforementioned problems, there has not been any work realizing super-resolution imaging of high-density PPIs in a cell at the sub-diffraction level.

Here, we develop a BiFC-PALM method dedicated for imaging the subcellular distribution and dynamics of individual interacting-protein pairs at high spatial-temporal resolution (Fig. 1c). The new method is applied to studying the interaction between EF-Tu and MreB (EFTu-MreB-PPIs) in *E. coli*. Both proteins are abundant so that if imaged by conventional fluorescence co-localization, the distribution and dynamics of the interacting pairs would be buried in the fluorescence background from non-interacting proteins and also masked by optical diffraction in the tiny bacterial cells. Using BiFC-PALM, we are able to localize individual EFTu-MreB-PPIs and reveal their specific spatial distribution and localization-dependent heterogonous dynamics as a subpopulation of total EF-Tu, providing important insights about their roles in bacterial cell wall synthesis. This is the first time that heterogonous dynamics of high-density interacting protein pairs is observed in the sub-diffraction cellular space.

## Results

### Development of BiFC-PALM fluorescent probe

**Figure 2:**
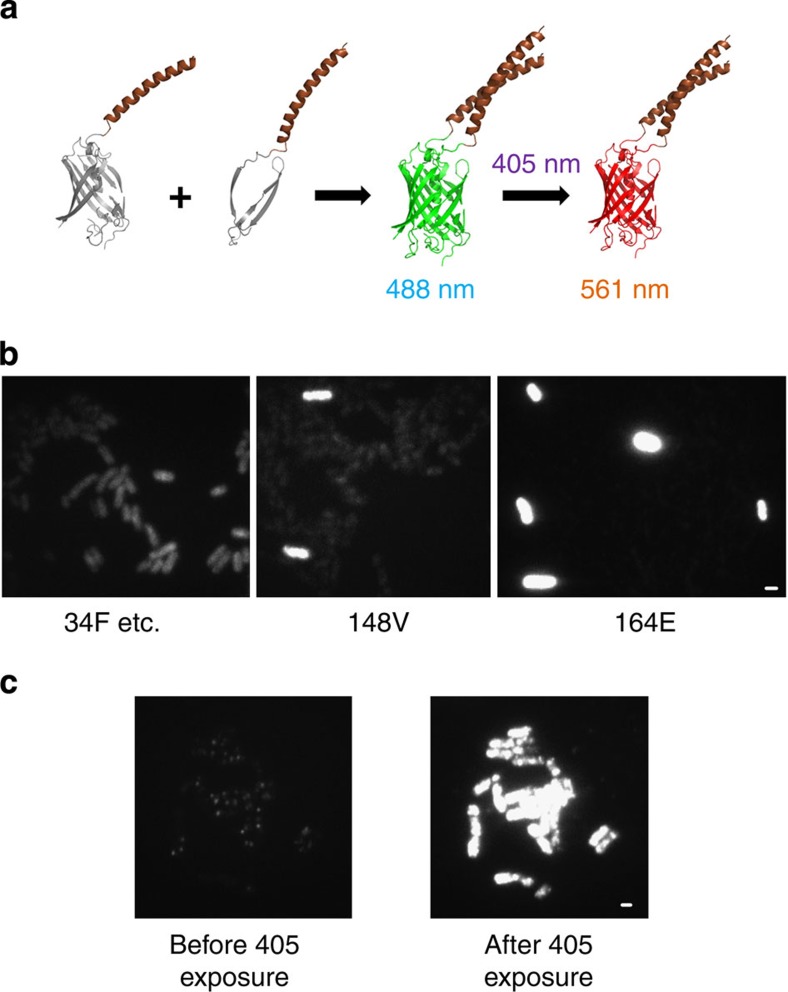
Construction and screening of complemented mEos3.2. (**a**) Schematic illustration of mEos3.2 complementation and its photoconversion. (**b**) Seven cleavage sites at different flexible loops of mEos3.2 (34F, 96E, 138K, 148V, 150D, 160A, 164E) yielded highly variable BiFC signal. Site 164E generated the highest fraction of bacteria cells with bright BiFC signal. (**c**) 405 nm irradiation converted 164E complemented mEos3.2 to the red form, which was excited by a 561-nm laser. Scale bar 1 μm.

BiFC-PALM requires a photoactivatable fluorescent protein, which can retain the ability of photoactivation and fluorescing when its two split fragments complement and refold (Fig. 2a). A photoswitchable fluorescent protein, Dronpa, has actually been tested for BiFC^12^, but its mediocre intensity contrast between the bright and dark states disqualifies Dronpa from being a super-resolution and single-molecule probe *in vivo*. Instead, we chose mEos3.2, a recently developed photoconvertible fluorescent protein^13^, as the BiFC-PALM probe. mEos3.2 is truly monomeric, which is crucial for BiFC. In addition, mEos3.2 demonstrates excellent performance in PALM imaging in terms of its brightness, maturation time and labelling density^13^.

A fluorescent protein may have multiple cleavage sites suitable for BiFC. We examined seven sites that are located on flexible loops of mEos3.2 (Fig. 2b). Each pair of split fragments (mEosN and mEosC) were fused to leucine zippers and expressed in *E. coli* to examine the complementation of mEosN and mEosC via specific leucine zipper formation. Among all seven cleavage sites, only 148V and 164E successfully generated BiFC fluorescence (Fig. 2b). Further, 164E showed much higher fraction of bacterial cells with BiFC signal than 148V, suggesting 164E as the optimal cleavage site for mEos3.2 (Supplementary Fig. 1a). The complementation efficiency of site 164E, defined as the intensity ratio between specific BiFC signal by leucine zippers and nonspecific BiFC signal through spontaneous complementation via mutated leucine zippers, was up to 12 (Supplementary Fig. 1), similar to that of Venus^11^, so far the most used fluorescent protein for BiFC.

We then confirmed if the complemented mEos3.2 is still photoconvertable. A 405-nm laser was used to convert complemented mEos3.2(164E) from green to red form, which was then excited by a 561-nm laser. Both *in vivo* and *in vitro* measurements showed that complemented mEos3.2(164E) not only retained its photoconvertability (Fig. 2c and Supplementary Movie 1) but also held similar photophysical properties to that of native mEos3.2 (Supplementary Fig. 1 and Supplementary Table 1). For a fluorophore used for STORM/PALM imaging, localization precision and photoactivation/photo-conversion rate are key parameters defining the spatial and temporal resolutions^14^. We therefore used these criteria to compare split mEos3.2(164E) with mEos3.2(148V), split Venus(155A) and split Dronpa(164E). Among the three fluorescent proteins, split mEos3.2(164E) and mEos3.2(148V) showed higher localization precision than split Venus(155A), whereas split Dronpa(164E) gave the least localization precision (Supplementary Fig. 2a). Regarding the photo-activation/photo-conversion rate, both mEos3.2(164E) and Dronpa(164E) were more sensitive to the activation illumination than mEos3.2(148 V). However, Dronpa(164E) showed a high spontaneous photo-activation rate, placing restrictions to its Nyquist spatial resolution (Supplementary Fig. 2b)^15^. In summary, we conclude that split mEos3.2(164E) is a better fluorescent protein for BiFC-PALM compared with split Venus(155A) and split Dronpa(164E).

### Super-resolution imaging of EFTu-MreB-PPIs using BiFC-PALM

BiFC-PALM based on mEos3.2 makes it possible to image the distribution of a certain PPI in a sub-diffraction regime, as a subpopulation of total protein of interest. To test-drive this new method, we chose abundant MreB and EF-Tu^16^,^17^ as a pair of model PPI. MreB is an actin cytoskeleton homologue involved in bacterial cell shape maintenance and chromosome segregation in rod-like cells^17^,^18^,^19^,^20^,^21^. Previous results have suggested that MreB may polymerize into helical structures along the internal membrane, extending longitudinally through the cell^21^,^22^,^23^. The extended filamentous structures of MreB were later examined by super-resolution imaging^24^,^25^. In addition, recent single-molecule tracking experiments suggest that MreB can form discrete patches that move slowly perpendicular to the longitudinal axis and direct insertion of newly synthesized peptidoglycan^24^,^25^,^26^,^27^,^28^. EF-Tu is one of the prokaryotic translation elongation factors. Although its main function is to deliver tRNA onto ribosomes, EF-Tu has been also found to have an important role in cell wall synthesis of *Bacillus subtilis (Bsu)*, through interaction with MreB^29^. Therefore, in order to fully understand how MreB and EF-Tu are coordinated in cell morphology maintenance, it is important to image MreB and EF-Tu as interacting pairs.

**Figure 3:**
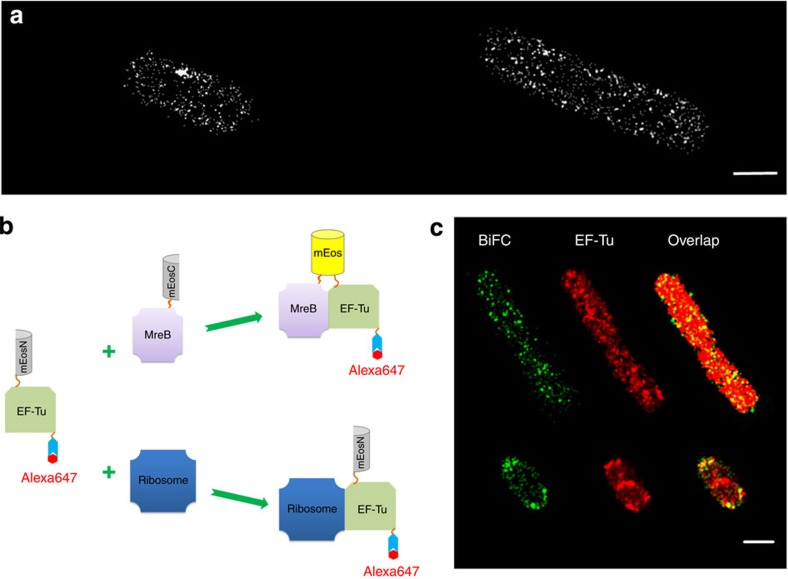
BiFC-PALM super-resolution imaging of EFTu-MreB-PPIs. (**a**) BiFC-PALM imaging of EFTu-MreB-PPIs in two fixed *E. coli* cells. (**b**) Schematic illustration indicating that EF-Tu can interact with multiple proteins in the cell. EF-Tu molecules that interact with MreB can be visualized by both Snap-Alexa647 (upper path) and complemented mEos3.2, whereas ET-Tu molecules that interact with other proteins or free EF-Tu can be visualized by Snap-Alexa647 (lower path); (**c**) EFTu-MreB-PPIs distribution obtained by BiFC-PALM (green) as a subpopulation of total EF-Tu labelled with Alexa647 (red) in two fixed cells. Scale bar, 1 μm.

We first used pull-down assay to confirm that MreB and EF-Tu also have specific interaction in *E. coli* (Supplementary Fig. 3), consistent with an interactome study of *E. coli* using mass spectrometry^1^. The detection and quantification of EFTu-MreB-PPIs in *E. coli* were obtained using BiFC-PALM of mEosN-EF-Tu and MreB-mEosC in fixed cells. The expression levels of both fusion proteins were much lower than their endogenous counterparts (Supplementary Fig. 4). The specificity of the BiFC signal was confirmed by two non-interacting pairs, EFTu-MreC and EFTu-MreD^1^, as well as a truncated EF-Tu with MreB (Supplementary Fig. 5)^30^. In the super-resolution images, the EFTu-MreB-PPIs seemed to exist in two forms, clusters and small dots, which did not seem to have distinct patterns (Fig. 3a and Supplementary Figs 6–8). We analysed 143 bacterial cells, among which 100 demonstrated rod shapes with normal aspect ratios (length/width ratio between 2.5 and 6), similar with the bacteria in Fig. 3a. We performed cluster analysis on 15 such type of bacteria (Supplementary Fig. 6). The pairwise distance distributions of all dots (Supplementary Fig. 6b) and clusters (Supplementary Fig. 6c) in 15 bacterial cells were similar, all approximating a generalized beta distribution^31^. This suggests that only a small fraction of EFTu-MreB-PPIs formed clusters, whereas the majority did not form higher-order structures and distributed rather randomly. The number of clusters in each bacterium was nearly proportional to the cell size with a density of ~80 clusters per μm^2^ (Supplementary Fig. 6d,e). The 15 bacterial cells were not only similar in cluster density and distance, but also similar in cluster area (Supplementary Fig. 6f,g).

Among the 143 bacterial cells, 40 were found to become oval (Supplementary Fig. 7), and 3 became elongated (Supplementary Fig. 8). Cluster analysis suggests that the EFTu-MreB-PPIs in neither oval-shaped nor elongated cells showed significant variation internally or externally compared with the normal cells in Supplementary Fig. 6. Nonetheless, it is interesting to note that the cluster density of oval-shaped bacterial cells was noticeably higher than that of both normal and elongated cells (Supplementary Figs 6–8e), implying a correlation between the bacteria phenotype and the density of EFTu-MreB-PPIs.

As EF-Tu has much higher copy number than MreB, we constructed mEosN-EF-Tu-Snap to see how EFTu-MreB-PPIs distribute as a subpopulation of total EF-Tu in the same cell (Fig. 3b,c and Supplementary Fig. 9). We analysed eight bacterial cells that co-expressed MreB-mEosC and mEosN-EFTu-Snap, in which all ectopically expressed EF-Tu could be independently observed by BG-Alexa647-labelled Snap-tag. We observed different patterns for EFTu-MreB-PPIs, including patch-like and polar localization (green in Fig. 3c and Supplementary Fig. 9a). Cluster analysis suggests that EFTu-MreB-PPIs in these cells were different from that in Supplementary Figs 6–8. For instance, the pairwise distance curves were highly variable and no longer a beta distribution (Supplementary Fig. 9b). The mean size of EFTu-MreB-PPI clusters (Supplementary Fig. 9f) was also noticeably larger than that in Supplementary Figs 6–8. The difference in EFTu-MreB-PPI parameters might be due to the perturbance from Snap-tag in mEosN-EFTu-Snap or the stress induced by electroporation. In spite of the difference, we note that the BiFC signal of EFTu-MreB-PPIs co-localized well with the EF-Tu-Snap signal, supporting that BiFC-PALM can detect the interacting pairs specifically.

We then used two-colour super-resolution imaging to evaluate the performance of co-localization approach for quantification of interactions between endogenous EF-Tu (EF-Tu-mEos2) and MreB (immuno-labelled MreB) in fixed cells (Supplementary Fig. 10). Unlike in *Bsu*^29^, the distribution of EF-Tu-mEos2 in *E*. *coli* did not show obviously growth-phase dependence (Supplementary Fig. 10a). Moreover, the difference between the even distribution of EF-Tu-mEos2 and patched distribution of BG-Alexa647-labelled EF-Tu (Supplementary Fig. 9a) might be again due to the perturbance from Snap-tag in mEosN-EFTu-Snap or the stress induced by electroporation. Unlike the high level of overlapping observed by conventional fluorescence imaging^29^, co-localization between EF-Tu and MreB seemed rather low in the super-resolution images (Supplementary Fig. 10b).

To obtain a more quantitative comparison on the co-localization levels between BiFC/EF-Tu (Fig. 3c and Supplementary Fig. 9a) and MreB/EF-Tu (Supplementary Fig. 10b), we performed a pixel-overlapping analysis and found that about 80% of EFTu-MreB-PPIs were overlapped with EF-Tu (BiFC on EF-Tu), significantly higher than that of MreB, which showed 40% overlapping with EF-Tu (MreB on EF-Tu), including both specific EFTu-MreB-PPIs and nonspecific overlap because of finite spatial resolution (Supplementary Fig. 10c). In summary, BiFC-PALM much outperformed the two-colour co-localization method in imaging of PPIs in a sub-diffraction space.

### BiFC-PALM single-molecule tracking of individual EFTu-MreB-PPIs

**Figure 4:**
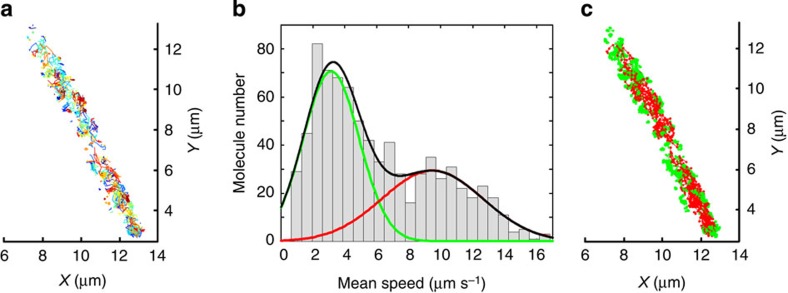
BiFC-PALM single-molecule tracking of individual EFTu-MreB-PPIs. (**a**) Two-dimensional trajectories of individual EFTu-MreB-PPIs in a live *E. coli* cell. The colour for each trajectory was randomly picked to distinguish nearby traces. (**b**) Mean speed histogram reveals that EFTu-MreB-PPIs had two populations with different mobility. Data were from three cells. (**c**) Spatial view of EFTu-MreB-PPIs with different mobility in a live *E. coli* cell. The red trajectories represent EFTu-MreB-PPIs that were moving faster than 6 μm s^−1^ and the slower ones are presented in green colour.

**Figure 5:**
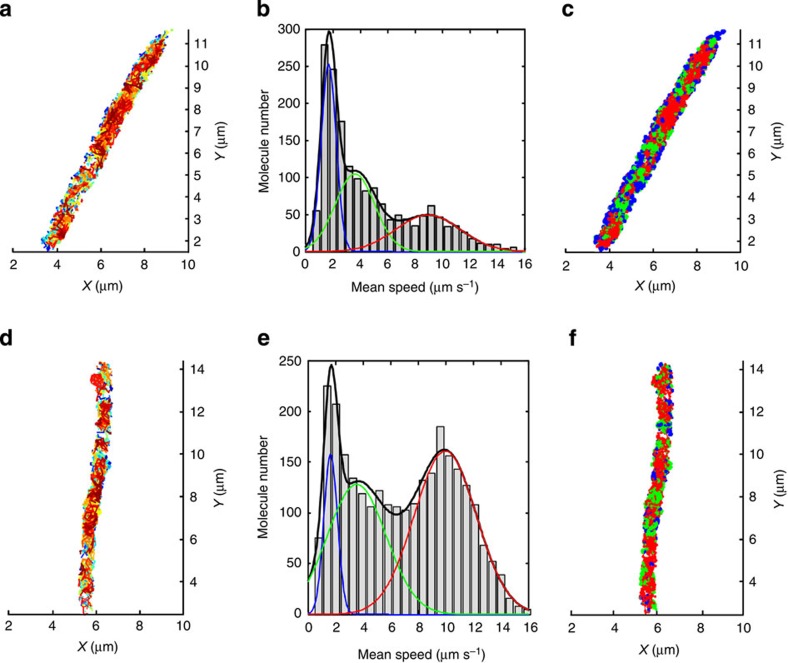
PALM single-molecule tracking of individual MreB and EF-Tu molecules. (**a**,**d**) Two-dimensional trajectories of individual MreB and EF-Tu in a live *E. coli* cell. (**b**,**e**) Mean speed histogram reveals that MreB and EF-Tu had three populations with different mobility. Triple-Gaussian fit (black) provides a low (blue), intermediate (green) and high (red) mobility population. Data were from three cells. (**c**,**f**) Spatial view of MreB and EF-Tu with different mobility in a live *E. coli* cell. The red trajectories represent molecules that were moving faster than 6 μm s^−1^, green for molecules faster than 2.5 μm s^−1^ and blue for the rest with low mobility.

BiFC has actually been used for single-molecule tracking of G-protein-coupled receptor dimers in mammalian cells, but that was not in a crowded, diffraction-limited cellular space^32^. We took the advantage of photo-controlled convertibility of split mEos3.2 to repeatedly convert and track minimal number of EFTu-MreB-PPIs in live *E. coli* cells using BiFC-PALM (Fig. 4a, Supplementary Fig. 11 and Supplementary Movie 2). Analysis of the single-molecule trajectories can provide information not only about the mobility of each molecule but also their spatial distribution (Supplementary Figs 12–14). For instance, the mean frame-to-frame speed histogram of all trajectories show that EFTu-MreB-PPIs had two populations with different mobility (Fig. 4b). Interestingly, spatial view of the two populations reveals clear dependence of molecule mobility on their locations, with slow-moving ones localizing near the cell periphery and faster ones within the cell (Fig. 4c). The two motility populations are also found to be related to the polymerization state of MreB because addition of MreB perturbing compound A22 was found to change the fractions of slow and fast mobility populations dramatically (Supplementary Fig 15). In contrast, separate super-resolution single-molecule tracking of MreB and EF-Tu dynamics reveals that both of them had three different mobility populations, which were also localization dependent (Fig. 5). It needs to note that the distribution of EFTu-MreB-PPIs (Fig. 4b) could also be fitted with a triple Gaussian function that is statistically equivalent to the double Gaussian fit at 5% level of significance, see Supplementary Fig. 16 for more discussion and the corresponding fractions of three mobility populations are given in Supplementary Table 2.

To further understand the role of EFTu-MreB interactions, we imaged the distribution of MreB alone in bacteria that expressed MreB-mEos3.2-Sandwich (MreBmEosSW) (Supplementary Fig. 17)^33^. The pairwise distance distribution of all MreB molecules indicates that majority of MreB formed aggregated states with a characteristic distance about ~900 nm between nearby clusters (Supplementary Fig. 17b), in contrast to the nearly even distribution of EFTu-MreB-PPIs (Supplementary Fig. 6b). It is also interesting to note that MreB seemed to organize differentially at different growth phases (Supplementary Fig. 17b). In addition, the median cluster area of MreB (Supplementary Fig. 17g) was noticeably larger than that of EFTu-MreB-PPIs (Supplementary Fig. 6g). These results imply that compared with the fraction of MreB in the EFTu-MreB complexes, the rest of MreB tend to form certain higher-order structures, and the interaction between MreB and EF-Tu may regulate each other’s states or functions, such as polymerization and cell wall synthesis. In fact, *E. coli* cells were found to gradually grow from a rod shape into a football-like shape after one round of division when they continuously expressed BiFC EFTu-MreB-PPIs (Supplementary Fig. 18b and Supplementary Movie 3), similar with the strain that overexpressed tethered MreB and EF-Tu via a linker peptide (Supplementary Fig. 18c). This is in striking contrast with the situation that when only MreB or MreB-mEosC was overexpressed, the bacterial cells tended to grow longer, consistent with previously reported observation^29^.

## Discussion

We have shown that the newly developed BiFC-PALM is able to reveal the form, distribution and dynamics of EFTu-MreB-PPIs. With the high spatial resolution and capability of quantification, BiFC-PALM study may provide new insights for the role of EFTu–MreB interactions.

MreB has been suggested to polymerize into short filaments underneath the bacterial inner membrane, where they dock cell-wall synthesis machinery. This is consistent with our super-resolution imaging of *E. coli* MreB, which formed patch-like domains that are mainly located at the cell periphery (Supplementary Fig. 17). Note that the states of MreB may highly depend on the states of targeted bacteria, including growth phases and external stress. Actually, when we performed live cell imaging of MreB, we often observed short and extended filaments moving very slowly across the longitude of the bacterium, consistent with the results reported by Reimold *et al.*^25^.

In *E. coli*, EF-Tu and MreB seem to have fairly low co-localization (Supplementary Fig. 10b) compared with that in *Bsu*^29^, which may be due to either largely increased spatial resolution in this study or intrinsic difference between *E. coli* and *Bsu*. BiFC-PALM imaging of EFTu-MreB-PPIs revealed distribution of interacting EFTu-MreB pairs as a subpopulation of total EF-Tu (Fig. 3c and Supplementary Fig. 9), and its specificity was confirmed by the high co-localization level between EFTu-MreB-PPIs and EF-Tu-Snap. Cluster analysis (Supplementary Figs 6g and 17g) suggests that binding of EF-Tu might prevent MreB from polymerizing into long filaments. Although it is believed to be mainly responsible for delivering tRNA to ribosomes, EF-Tu has a much higher copy number than ribosome. In echo of this fact, a study in *Bsu* suggested that EF-Tu has an additional role in cell shape maintenance through interaction with MreB^29^. Intensity analysis of Fig. 3c estimated that MreB-bound EF-Tu, that is, EFTu-MreB-PPIs, was about 16% of the total EF-Tu (Supplementary Table 2). The estimation was for ectopically expressed proteins. If we assume that the fraction holds true for endogenous EF-Tu and MreB, there would be ~15,000 EFTu-MreB-PPIs given the copy number of EF-Tu as 90,000 (ref. 16), meaning that 50% of MreB^17^ interacted with EF-Tu (Supplementary Table 2).

In addition to its spatial distribution, the dynamics of MreB has also been proven essential for cell wall synthesis. The slowly circumferential motion of MreB filaments driven by insertion of new peptidoglycan acts back to result in uniform cell wall insertion, which enables the cell to maintain its rod shape^26^,^27^,^28^. The dynamics of MreB filaments is found to relate to its ATPase activity, as a point mutation in phosphate 2 motif of MreB reduces the mobility of the filaments and causes a severe defect in cell morphology^17^,^34^. Our super-resolution single-molecule tracking in live *E. coli* cells reveals location-dependent heterogonous dynamics for EF-Tu (Fig. 5f), MreB (Fig. 5c) and EFTu-MreB-PPIs (Fig. 4c), which are also dependent on the polymerization states of MreB (Supplementary Fig 15). Figure 5b,e show that about 50% of EF-Tu belonged to the high-mobility population, whereas the fraction for MreB was only 29% (Supplementary Table 2). This is consistent with the previously reported low copy number of MreB (~1,500) in *E. coli* cytosol^16^, compared with the total of 17,000–40,000 (ref. 17). In contrast, the low-mobility populations (*v*=1.7 μm s^−1^) were mainly located at the cell periphery. Taking account of the instant speed caused by localization error, the low-mobility population moved actually very slowly. This was proven by a time lapse tracking of MreB at a much longer interval (2.5 s), which showed these molecules actually moved at ~15 nm s^−1^ perpendicular to the long axis (Supplementary Movie 4), similar with the measurements of MreB filaments in *Bsu*^24^,^25^,^26^,^27^ and in *E. coli*^25^,^28^. Note that the low-mobility fraction of EFTu-MreB-PPIs was only 4.3%, suggesting EFTu-bound MreB and free MreB may be different in states and functions (Supplementary Table 2). The intermediate mobility populations were also mainly located at the cell periphery with some large patches crossing the cell. The localization suggests that these intermediate mobility populations were likely membrane-associated units, but their functions and polymerization states are not known.

The effect of EF-Tu on MreB was also showed by the football-shape phenotype of *E. coli* strains that overexpressed BiFC EFTu-MreB or tethered EFTu-MreB (Supplementary Fig. 18). This phenotype has also been observed for strains that have either MreB knocked out^20^,^23^ or disrupted MreB polymerization using small-molecule A22 (refs 17, 20). The cell rounding is therefore thought to be caused by the reduction of polymerized MreB, which not only directs cell wall synthesis, but also contributes to the mechanical rigidity of a cell^35^. Therefore, overexpression of BiFC EFTu-MreB would reduce the amount of free MreB molecules that are available for polymerization and thus cause the cell to grow round (Supplementary Fig. 19). Unfortunately, in contrast to the good understanding of actin, essential structural information about the polymerized MreB and the interaction between MreB and EF-Tu is still missing^23^,^36^. To fully understand the role of EFTu-MreB interactions, further experiments are needed in structural biology, electron microscopy and single-molecule fluorescence assays.

In summary, we developed a new technique named BiFC-PALM for studying PPIs with high specificity and spatial-temporal resolution in live cells. Its application in live bacterial cells revealed interesting distribution and heterogonous dynamics of EFTu-MreB-PPIs, which would otherwise be buried in the fluorescent background from the non-interacting proteins using conventional fluorescence imaging. For both MreB and EF-Tu, in contrast to their whole population, the interacting-protein pairs exhibited different distribution and dynamics, implying mutual regulations via the interaction. Future development of BiFC-PALM would lie in the direction to multi-colour for detection of multiple PPIs as well as expanded application in eukaryotic cells (Supplementary Fig. 20).

## Methods

### Plasmids and strain construction

The mEos3.2 sequence was kindly provided by Pingyong Xu (Institute of Biophysics, Chinese Academy of Sciences), and the leucine zipper sequence was purchased from Invitrogen. For all seven tested cleavage sites, 14 fragments were cloned from the mEos3.2 template using corresponding primers synthesized by Invitrogen, and fused to leucine zippers via flexible linkers (GGSGSG for mEosN-zipper and GGSG for Zipper-mEosC) by two-step PCR. mEos3.2N-Zipper was inserted into petduet-1 vector (Novagen) at restriction endonuclease multi-clone site *Nco*I/*Eco*RI and zipper-mEos3.2C at a multi-clone site *Bgl*II/*Xho*I. BiFC-Dronpa and BiFC-Venus were constructed similarly with split mEos3.2(164E). Zipper-mEos3.2C with triple point mutations (K13E, E18K, E25K) and (K6E, K13E, E18K) were constructed similarly. The EF-Tu-mEos2 and MreB-mEosC strains were all created by lambda red recombination based on strain BW25993. The Snap sequence was cloned from Psnap tag vector (New England Biolabs). For mEosN-EF-Tu plasmid, EF-Tu was cloned from bacteria genome and fused to mEosN via a flexible linker (GGSGSG), mEosN-EFTuΔNΔC was constructed similarly. The fusion PCR fragments were then inserted into pacycduet-1 vector (Novagen). Plasmids of MreB-mEosC, MreC-mEosC and MreD were all similarly prepared. For mEosN-EF-Tu-Snap plasmid, the Snap sequence was fused to mEosN-EF-Tu sequence and cloned into pbadmyc-hisA vector *Pst*I/*Eco*RI site. To track the single-molecule movement of MreB and EF-Tu, two plasmids: pet28amreBmEos3.2sandwich and pet28amEos3.2tufB were constructed. pet28amreBmEos3.2sandwich was constructed referring to a MreB–RFP SW method by Bendezú *et al.*^33^, but replaced red fluorescent protein (RFP) with mEos3.2. The insertion site was between G228 and D229 of MreB. Flexible linkers SGS and SGAPG were adopted. Pet28amEos3.2tufB was constructed similar as pet28amEosNtufB. For BiFC in HeLa cells, mEos3.2N-Zipper was inserted into pcDNA3.1(+)vector (Novagen) at multi-clone site *Nhe*I/*Bam*HI and zipper-mEos3.2C at a multi-clone site *Nhe*I/*Bam*HI. Zipper-mEos3.2C with triple point mutations (K13E, E18K, E25K) was constructed similarly.

### Characterization of BiFC efficiency and specificity

Plasmids were transformed into *E. coli* strain Bl21(de3) (Transgene) and a single positive colony was picked and inoculated overnight. The culture was amplified 1:100 into Luria Broth medium, induced with 200 μM isopropyl-β-D-thiogalactoside (IPTG) for 3 h at 30 °C, then collected and washed in filtered phosphate-buffered salt solution (PBS, pH7.4) for five times. The cells were then loaded into flow chamber pre-coated with polylysine (Sigma) and checked for fluorescence under an inverted fluorescence microscope with 488 nm irradiation.

To test the specificity of BiFC, two mutated zipper-mEos3.2C were separately induced, and their BiFC signal was compared with the 164E strain at different culture duration using a plate reader (Molecular Devices) with 480 nm excitation. The optical density (OD(600)) readings of the culture were used to normalize the BiFC fluorescence signal. To further confirm that mEosN-zipper and Zipper-mEosC were expressed at a comparable level in different strains, 10 μl of each culture was lysed and loaded onto a SDS–PAGE. The absorption and emission spectra of complemented mEos3.2 were also measured using a plate reader.

### Characterization of BiFC-mEos3.2

Split mEos3.2(164E) was compared with split mEos3.2(148 V), split Venus(155A) and split Dronpa(164E). All split fragments were complemented using leucine zippers via a flexible linker. The complemented fluorescent proteins were bound to a coverslip for all measurements. Split mEos3.2 was excited by a 561-nm laser (1 kW cm^−2^) and split Venus and Dronpa were excited by a 488-nm laser with the same laser power density (700 W cm^−2^). We collected the data at 60 Hz until all molecules were bleached. For each condition, data of at least three regions of interest were collected. Localization precision was determined as previously described^37^,^38^. To measure the photo-activation/photo-conversion rate *k*_on_, both activation and excitation lasers were kept on and the number of fluorescent spots were counted for each frame. Note that for any molecules that stayed or showed up multiple times were only counted once for their first appearance. A single exponential fit of the molecule counts as a function of time provides *k*_on_. *k*_on_ was also measured as a function of the power density of the 405 nm activation laser.

### Verification of MreB and EF-Tu interaction in *E. coli*

Snap-tagged MreB strain was generated by lambda red recombination. 50 ml cultures (OD(600)=0.5) were harvested and lysed. Cleared cell lysates were incubated for different time with 100 μl Snap capture beads (NEB) to pull down MreB interacting proteins. The specific interaction between MreB and EF-Tu was then verified by western blotting using an EF-Tu antibody (Hucult Biotech).

### MreB-EFTu BiFC specificity control

The 48–367 amino-acid region of *E. coli* EF-Tu was cloned, fused with mEosN with a flexible linker, cloned into pacycduet-1 vector. This mEosN-EFTuΔNΔC vector was transformed into Bl21(de3) competent cells with MreB-mEosC vector and induced by IPTG for 1 h, whereas another strain expressing mEosN-EFTu and MreB-mEosC was used as a control. SDS–PAGE was used to detect the expression level of mEosN-EFTuΔNΔC and mEosN-EFTu. For MreB-mEosC, western blotting was performed and a rabbit anti-MreB antibody was used to detect its expression level.

### Sample preparation for super-resolution imaging of *E. coli*

Generally, *E. coli* culture was harvested and washed in filtered PBS (pH7.4) for three times. The cells were then fixed with 4% paraformaldehyde for 15 min, washed three times by PBS buffer, and injected into a flow chamber pre-coated with polylysine for 30 min. Coverslips (Fisher 24 × 50) and slides were cleaned using Piranha solution (30% H_2_O_2_:98% H_2_SO_4_=1:3 at 90 °C for 30 min).

### Super-resolution co-localization imaging of MreB and EF-Tu

Endogenous MreB was immuno-labelled in EF-Tu-mEos2 strain to image co-localization between the two proteins in a single cell. The primary antibody for MreB was rabbit polyclonal antibody (Invitrogen) and the secondary antibody was goat anti-rabbit IgG (Jackson) tagged with Alexa647. The two-colour imaging was sequentially performed, with Alexa647 first being imaged by STORM. GLOX was added to protect Alexa647 from photobleaching, and β-mercaptoethanol to promote photoswitching^14^. A continuous constant 640 nm irradiation (~4 kW cm^−2^) was used in STORM imaging without 405 nm irradiation. mEos2 channel was collected with a continuous constant 561 nm irradiation (~2 kW cm^−2^) and a continuous 405 nm laser, which was slowly adjusted for optimal photoconversion rates. 100 nm Tetraspeck beads (Invitrogen) were used as fiducial markers for drift correction and alignment between the two channels.

### Super-resolution imaging of EFTu-MreB-PPIs in *E. coli* cells

Pacycduet-mEosN-tufB or pbad-mEosN-tufB-Snap was transformed into MreB-mEosC strain. When OD(600) of the culture reached 0.4–0.6, 200 μM (final concentration) IPTG was added to induce the expression of BiFC fragments for 1.5 h at 30 °C. Snap was labelled by BG-Alexa647. Briefly, BG-alexa647 (NEB) was shocked in to interact with the Snap tag for 1 h, followed by three times of PBS wash. The single- or two-colour super-resolution imaging was performed as aforementioned.

### Sample preparation for single-molecule tracking in *E. coli*

The *E. coli* transformation was done similarly with the fixed cell imaging except that the culture was inoculated into 3 ml M9 medium (50 × amino acids, 100 × vitamins) in a shaker at 37 °C overnight. The culture was amplified 1:100 into 8 ml M9 medium (50 × amino acids, 100 × vitamins)+0.4% glucose. When OD(600) reached 0.4–0.6, 200 μM (final concentration) IPTG was added to induce the expression of BiFC fragments for 1.5 h at 30 °C. The cells were then collected, washed and re-suspended in M9 medium (50 × amino acids)+0.4% glucose. The samples were loaded between a coverslip and a 3% low melting temperature agarose gel pad, so the bacteria were immobilized well for long-term live cell imaging. When A22 was used, a final concentration of 10 μg ml^−1^ was added 1 h before the bacteria was collected or directly into the imaging buffer for imaging experiments.

### Phenotype

We constructed three strains that express (MreB+mEosN-EFTu), (MreB-mEosC+mEosN-EFTu) and peptide linker-tethered MreB and EF-Tu, respectively. Strain preparation was similar as above. The induction time for (MreB+mEosN-EFTu) and (MreB-mEosC+mEosN-EFTu) was 6 h. The induction time for peptide linker-tethered MreB and EF-Tu was shortened to 1 h.

### Single-molecule tracking of EFTu-MreB-PPIs in *E. coli*

A 405-nm laser (~0.6 W cm^−2^) was pulsed for 20 ms every 3 s to convert the mEos3.2 molecules, and a continuous 561 nm laser irradiation (100 W cm^−2^) was used to track single molecules at 50 Hz.

### Optical setup for BiFC-PALM

BiFC-PALM and STORM/PALM imaging were done using a Nikon TiE inverted microscope equipped with a × 100 oil objective (NIKON, PLAN APO, 1.49 numerical aperture) and Andor-897 EMCCD (Andor). A 405-nm laser (Coherent, 100 mW), 488-nm laser (Coherent, 100 mW), 561-nm laser (Coherent, 50 mW) and 640-nm laser (Coherent, 100 mW) were used to either photoconvert or excite the fluorophores. For the two-colour imaging of mEos3.2 and Alexa647, a polychroic mirror set (Di01-R405/488/561/635-Dichroic and FF01-446/523/600/677-Emission) was used. The lasers were modulated by an acousto-optic tunable filtre (AA Opto Electronic) and the beam width was expanded fivefold and focused at the back focal plane of the objective. Laser pulses were generated by a set of mechanical shutters controlled by home-written Labview scripts.

### STORM/PALM data analysis

Super-resolution image reconstruction was performed using Insight3 software, generously provided by Dr Bo Huang (University of California, San Francisco). Post data analysis such as drift correction, chromatic correction and binning were done using home-written Matlab scripts.

### Data analysis for BiFC-PALM single-molecule tracking

The BiFC-PALM single-molecule data were analysed using a Matlab-based GUI programme Fiesta^39^. Molecules were identified and molecule trajectories were connected based on user-defined tracking algorithms. All molecule trajectories were analysed using home-written Matlab codes. Briefly, molecules that lasted less than six frames (0.12 s) were abandoned. The mean frame speed of survived molecules was calculated. Mean-squared displacement (MSD) was calculated based on a two-dimensional diffusion model (MSD=4*Dt*+*b*), where *D* represents the diffusion coefficient and *b* reflects the localization uncertainty^40^.

### mEos3.2 BiFC in living HeLa cells

Transfection of mEos3.2-N-Zipper and mEos3.2-C-Zipper or mEos3.2-C mutated zipper fusion constructs was carried out using Lipofectamine 2000 (Invitrogen) on HeLa cells cultured on 0.17-mm thick bottom glass dishes. Images were first acquired through green fluorescent protein (excitation filtre: 485/20, dichroic mirror: 410/504/582/669, emission filtre: 440/521/607/700, Semrock) and RFP (excitation filtre: 560/25, dichroic mirror: 410/504/582/669, emission filtre: 440/521/607/700, Semrock) channels. To test the photo-conversion capability of mEos3.2-BiFC-Zipper complexes, the cells were illuminated with ultraviolet light (excitation filtre: 387/11, Semrock) for 10 s and imaged with RFP channel again. All images were taken with 500 ms exposure time.

## Author contributions

Z.L. and Y.S. designed and performed experiments. D.X. designed mEos3.2 split sites and nonspecific control. X.K., J.Z., H.S. and Y.T. constructed plasmids. X.K., J.Z. performed nonspecific control experiments. Q.S., Z.L. and B.X. collected the STORM/PALM data and performed data analysis. S.W. and B.X. measured the complemented mEos3.2 spectrum. Y.Z. performed the co-localization analysis. Y.S. and Z.L wrote the manuscript.

## Additional information

**How to cite this article:** Liu, Z. *et al.* Super-resolution imaging and tracking of protein–protein interactions in sub-diffraction cellular space. *Nat. Commun.* 5:4443 doi: 10.1038/ncomms5443 (2014).

## Supplementary Material

Supplementary Information Supplementary Figures 1-20 and Supplementary Tables 1-3

Supplementary Movie 1 Photoconversion of leucine zipper induced complemented mEos3.2 using 405nm laser pulses to photoconvert and 561nm laser to excite the converted mEos3.2 fluorescent protein. The movie is sped up 2-fold and played at 20 fps.

Supplementary Movie 2 BiFC-PALM single molecule tracking of individual EFTu-MreB-PPIs in a live *E. coli* cell. The movie is slowed down by 2-fold and played at 20 fps.

Supplementary Movie 3 *E. coli* cells over-expressing EFTu-MreB-PPIs grew into football-like shape gradually.

Supplementary Movie 4 PALM single molecule tracking of individual MreB in a live *E. coli* cell with a long time interval of 2.5s. The movie is sped up by 80-fold and played at 20 fps.

## Figures and Tables

**Figure 1:**
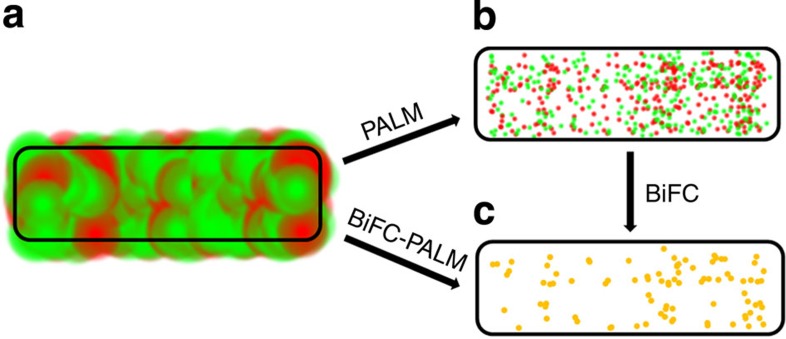
Comparison between two-colour STORM/PALM and BiFC-PALM. (**a**) Optical diffraction and fluorescent background from non-interacting proteins make it difficult to image specific protein–protein interactions. The red and green spots are the point spread functions of individual protein A and protein B molecules, respectively. The spatial resolution is about 200 nm. (**b**) Two-colour STORM/PALM co-localization imaging shows uncertainty on overlapping non-interacting molecules. The red and green spots are the single-molecule localizations of individual protein A and protein B molecules, respectively. The spatial resolution is about 20 nm. The high density of both proteins results in large uncertainty for identification of interacting protein pairs by co-localization. (**c**) BiFC-PALM can locate specific interacting pairs with high spatial resolution, given almost zero background from non-interacting molecules. The yellow spots are the single-molecule localizations of interacting pairs of protein A and protein B molecules. The spatial resolution is about 20 nm.
