# Supplementary material for: Super-resolution imaging and tracking of protein–protein interactions in sub-diffraction cellular space
Source: Nat Commun. 2014 Jul 17;5:4443. doi: 10.1038/ncomms5443 (PMC4109008; doi:10.1038/ncomms5443)
Supplement: Supplementary Information — Supplementary Figures 1-20 and Supplementary Tables 1-3 [file ncomms5443-s1.pdf]

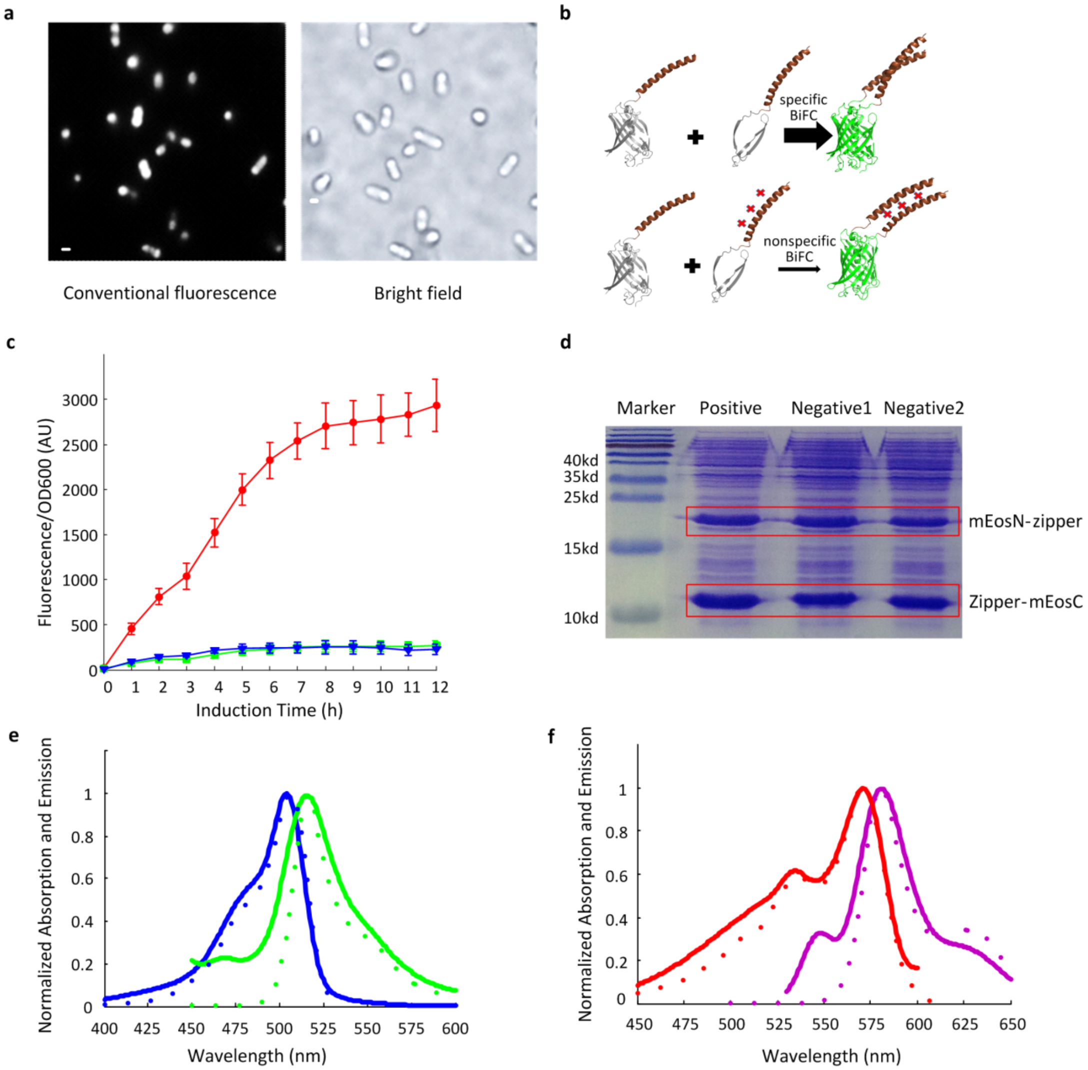

Supplementary Figure 1 | Characterization of complemented mEos3.2(164E). **(a)** Fluorescence and bright field images showing that nearly 100% bacterial cells that expressed mEos3.2(164E) had BiFC signal, scale bar 1 μm.; **(b)** Schematic illustration of specific BiFC through leucine zippers and non-specific BiFC through mutated leucine zippers; **(c)** Normalized BiFC signal as a function of induction time. Red: leucine zippers; Blue&green: mutated leucine zippers. The ratio between red and blue/green at the saturation phase provides the BiFC complementation efficiency about 12; **(d)** SDS-PAGE showed similar expression levels of mEosN-zipper and Zipper-mEosC for both strains with native and mutated leucine zippers; **(e)** The absorption and emission spectra of complemented mEos3.2 in its green form (solid), almost identical to that of native mEos3.2 (dashed); **(f)** Photoconverted complemented mEos3.2 (solid) mimics the spectra of native mEos3.2 (dashed).

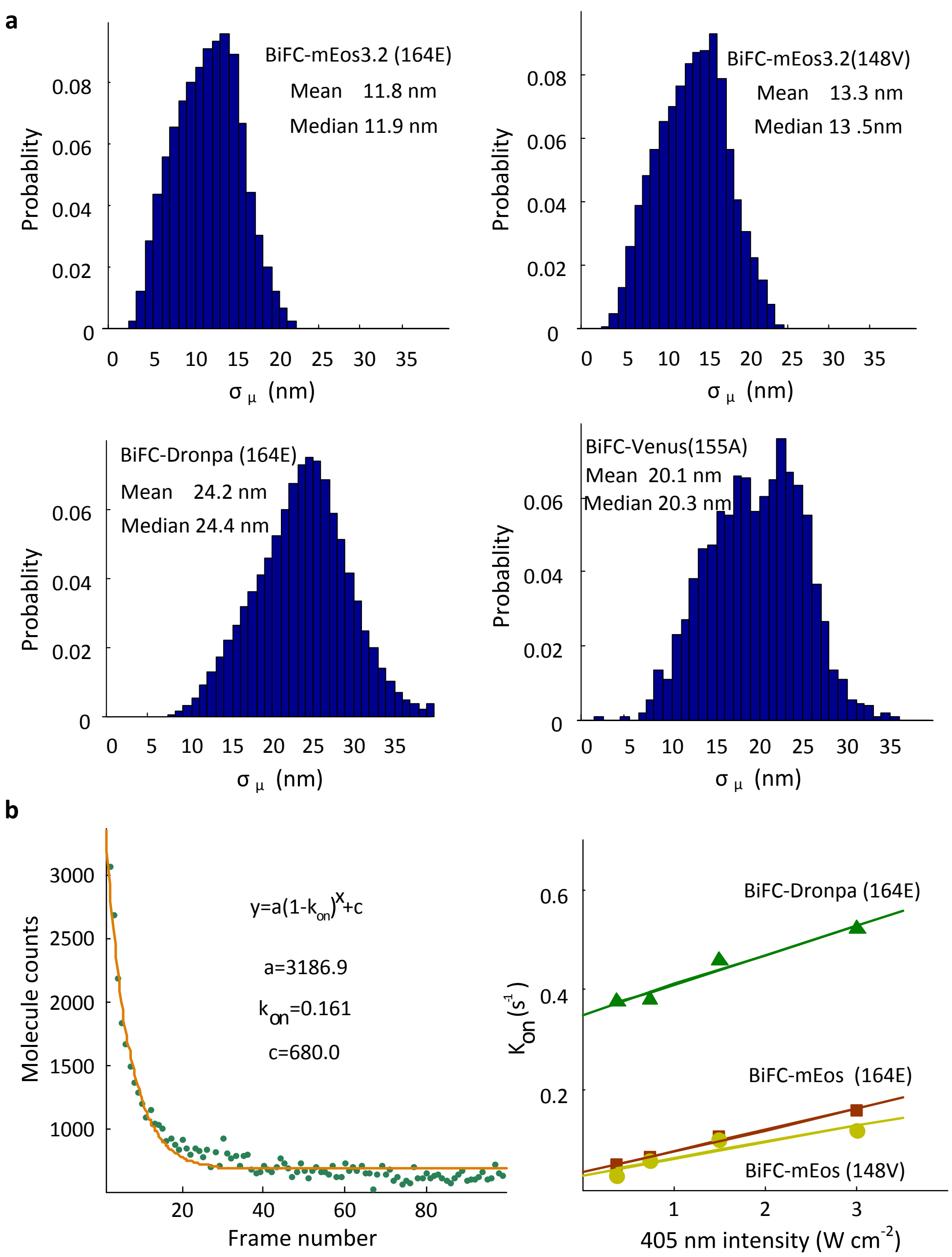

Supplementary Figure 2 | Characterization of split mEos3.2(164E) through comparisons with split mEos3.2(148V), split Venus(155A), and split Dronpa(164E). (a) Localization precision of different complemented fluorescent proteins; (b) Comparison of the photo-activation/photo-conversion rate k_{on} of different split fluorescent proteins. Left panel: k_{on} measured by fitting the number of activated fluorescent molecules observed in each frame; Right panel: Dependence of k_{on} on activation laser power density.

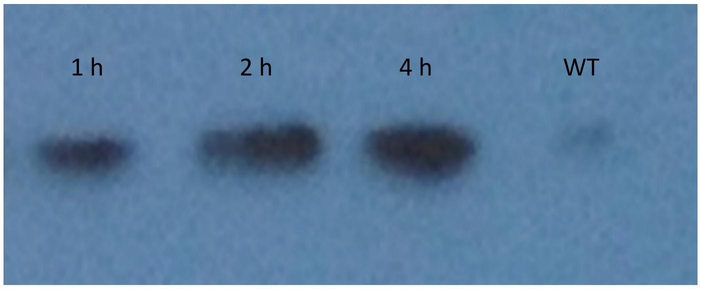

Supplementary Figure 3 | Verification of MreB and EF-Tu interaction through pull-down assay and western blotting. 50ml Snap-tagged MreB strain was cultured to OD(600)~0.5, harvested, and lysed. The supernatant was incubated for 1h, 2h and 4h (lanes #1-3), respectively, with 100μL Snap capture-beads (NEB) to pull down MreB interacting proteins. The specific interaction between MreB and EF-Tu was then verified by western blotting using an EF-Tu antibody. A wild type BW25993 cell lysate was incubated for 4 hours with Snap-capture beads as the specificity control (lane #4).

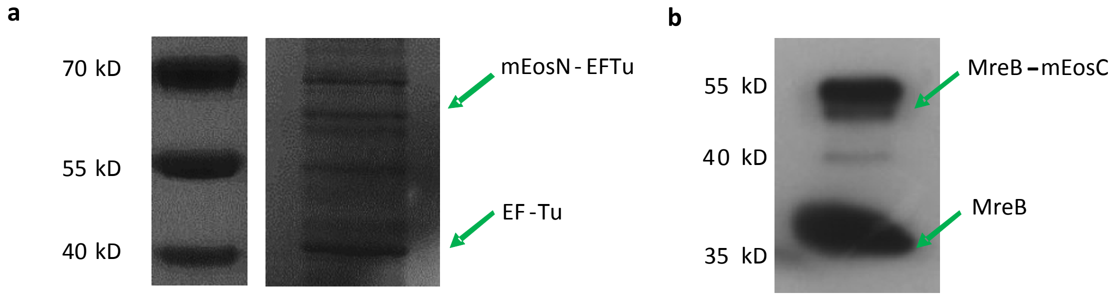

Supplementary Figure 4 | Quantification of the expression levels of fusion proteins versus the endogenous proteins. (a) mEosN-EF-Tu versus the endogenous EF-Tu determined by SDS-PAGE; (b) MreB-mEosC versus the endogenous MreB quantified by Western blotting.

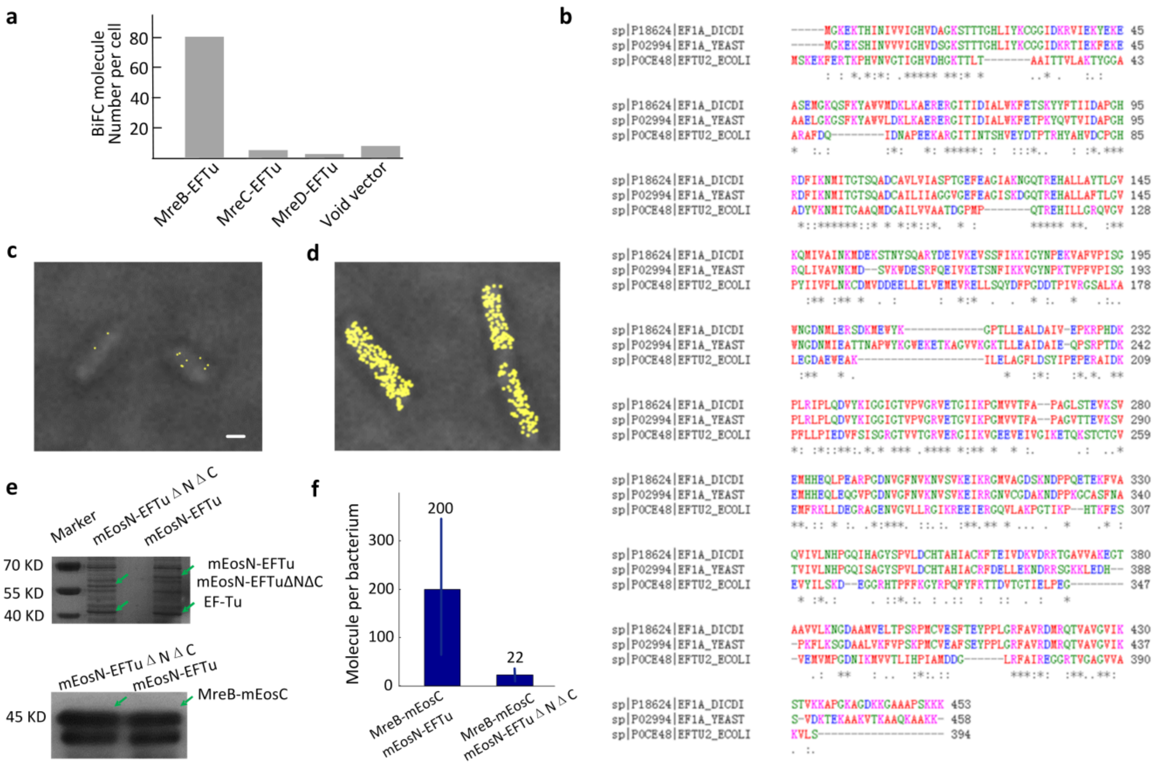

Supplementary Figure 5 | Characterization of BiFC specificity using non-interacting pairs, EFTu-MreC and EFTu-MreD, as well as a truncated EF-Tu (mEosN-EFTuΔNΔC) with MreB. (a) Specificity of EFTu-MreB BiFC confirmed by negative controls of MreC and MreD; (b-f) Specificity of EFTu-MreB BiFC quantified by mEosN-EFTuΔNΔC. (b) Sequence alignment between EF-Tu and its eukaryotic homolog EF1a; (c) Non-specific BiFC signal in bacterial cells co-expressing mEosN-EFTuΔNΔC and MreB-mEosC, scale bar 1 μm.; (d) Specific BiFC signal in bacterial cells co-expressing mEosN-EFTu and MreB-mEosC; (e) The expression level of mEosN-EFTuΔNΔC was similar with mEosN-EFTu, and the expression levels of MreB-mEosC were similar in both strains; (f) Quantification of BiFC counts per bacterium in 20 bacterial cells with specific EFTu-MreB BiFC and 11 with non-specific BiFC between EFTuΔNΔC and MreB, the error bar is standard deviation.

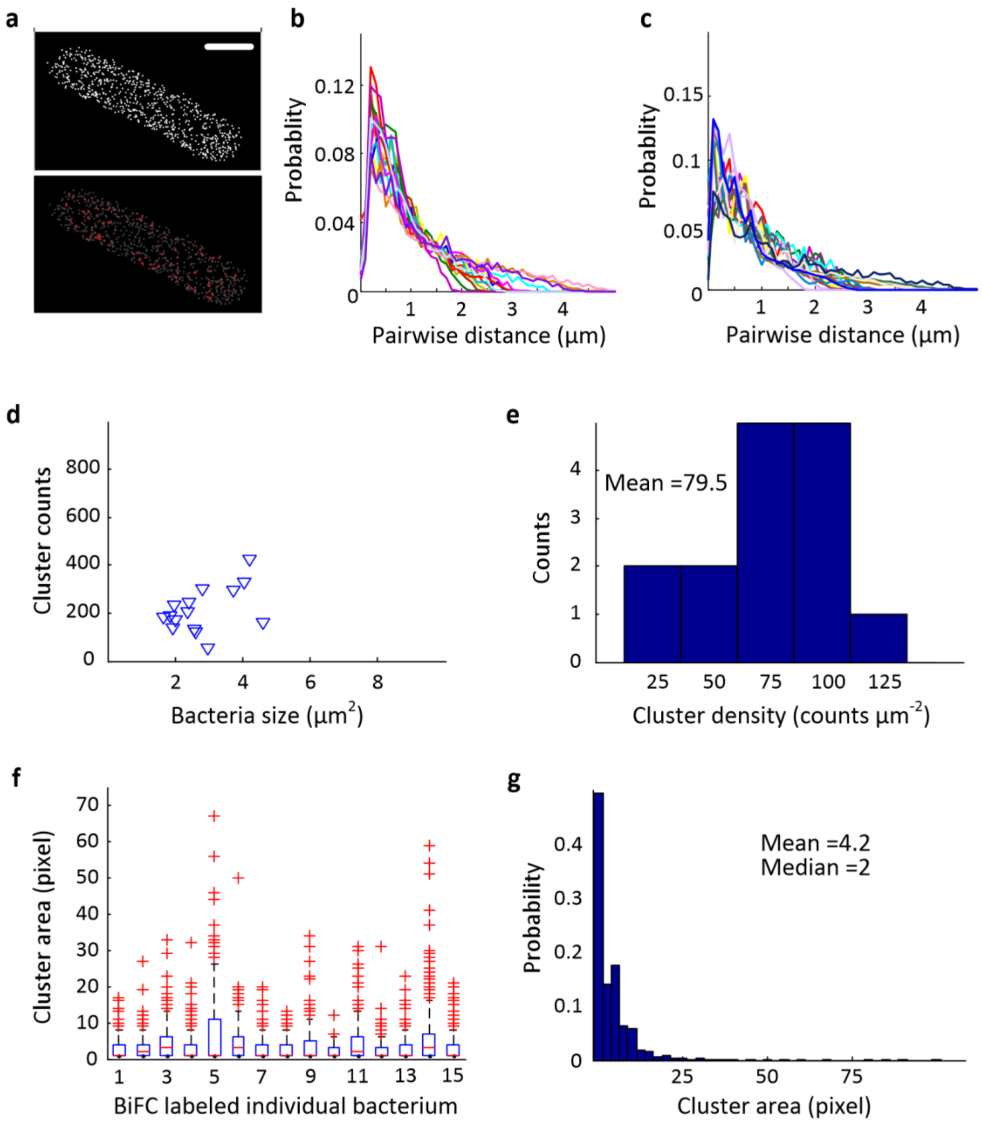

Supplementary Figure 6 | Cluster analysis of EFTu-MreB-PPI super-resolution images of rod shape bacterial cells with normal aspect ratios, similar with the one in Fig.2a. (a) Upper: EFTu-MreB-PPIs appeared like dots and clusters in the super-resolution images, and Lower: clusters were mapped out using a Gaussian filter and ImageJ plugin. The threshold was set so that single pixeled dots were removed; (b-g) Cluster analysis of the super-resolution images. (b) Pairwise distance distribution of all dots in the upper panel of (a); (c) Pairwise distance distribution of all clusters in the lower panel of (a); (d) Number of clusters in each bacterium as a function of the cell size; (e) Cluster density distribution of all 15 bacteria; (f) Median cluster area of each bacterium; (g) Cluster area distribution of all 15 bacteria. Scale bar 1 μm.

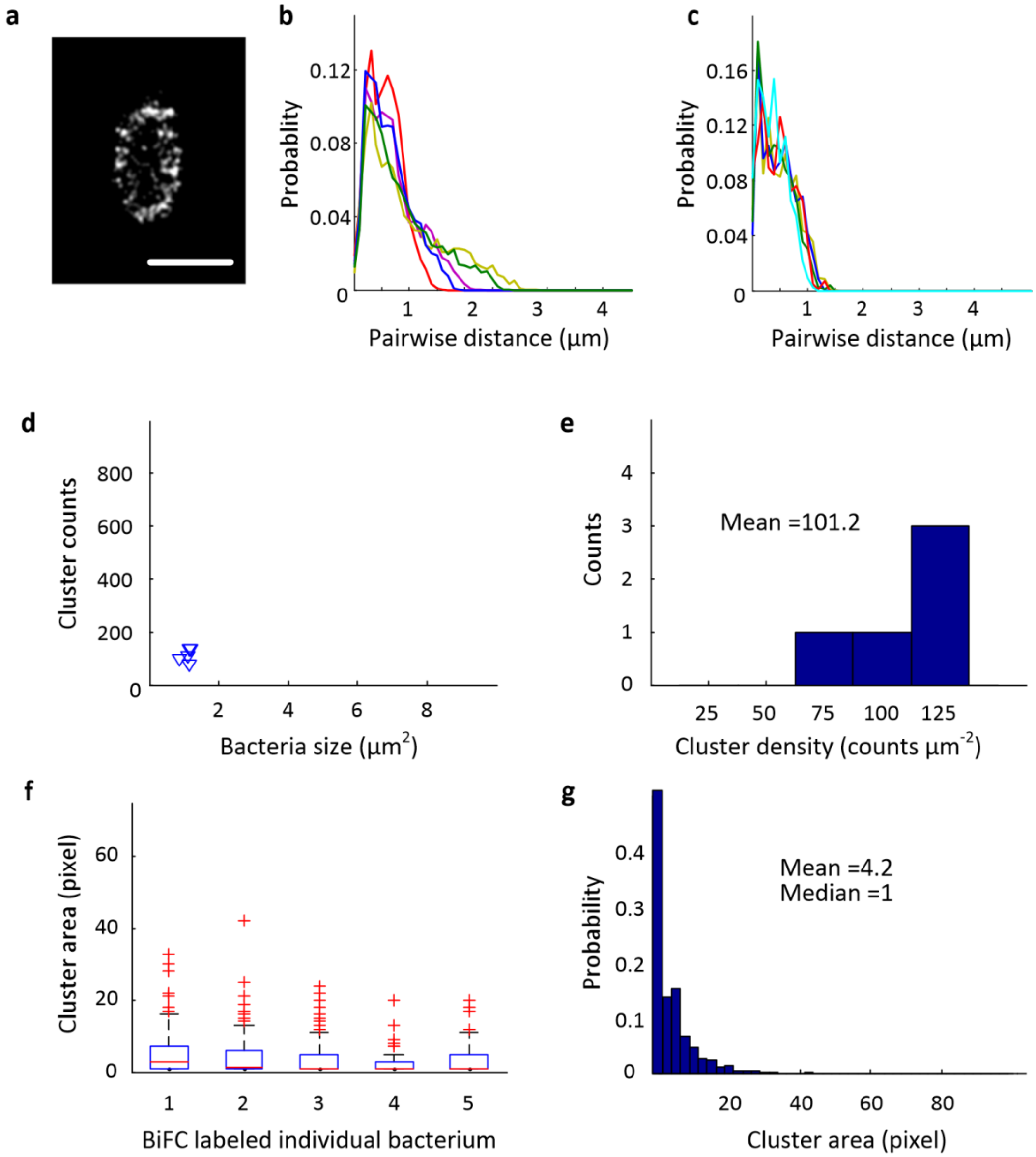

Supplementary Figure 7 | Cluster analysis of EFTu-MreB-PPI super-resolution images of oval shaped bacterial cells. (a) One bacterial cell demonstrated an oval shape; (b-g) Cluster analysis of the super-resolution images. (b) Pairwise distance distribution of all dots in the bacteria; (c) Pairwise distance distribution of all clusters in the bacteria; (d) Number of clusters in each bacterium as a function of the cell size; (e) Cluster density distribution of all 5 bacteria; (f) Median cluster area of each bacterium; (g) Cluster area distribution of all 5 bacteria. Scale bar 1 μm.

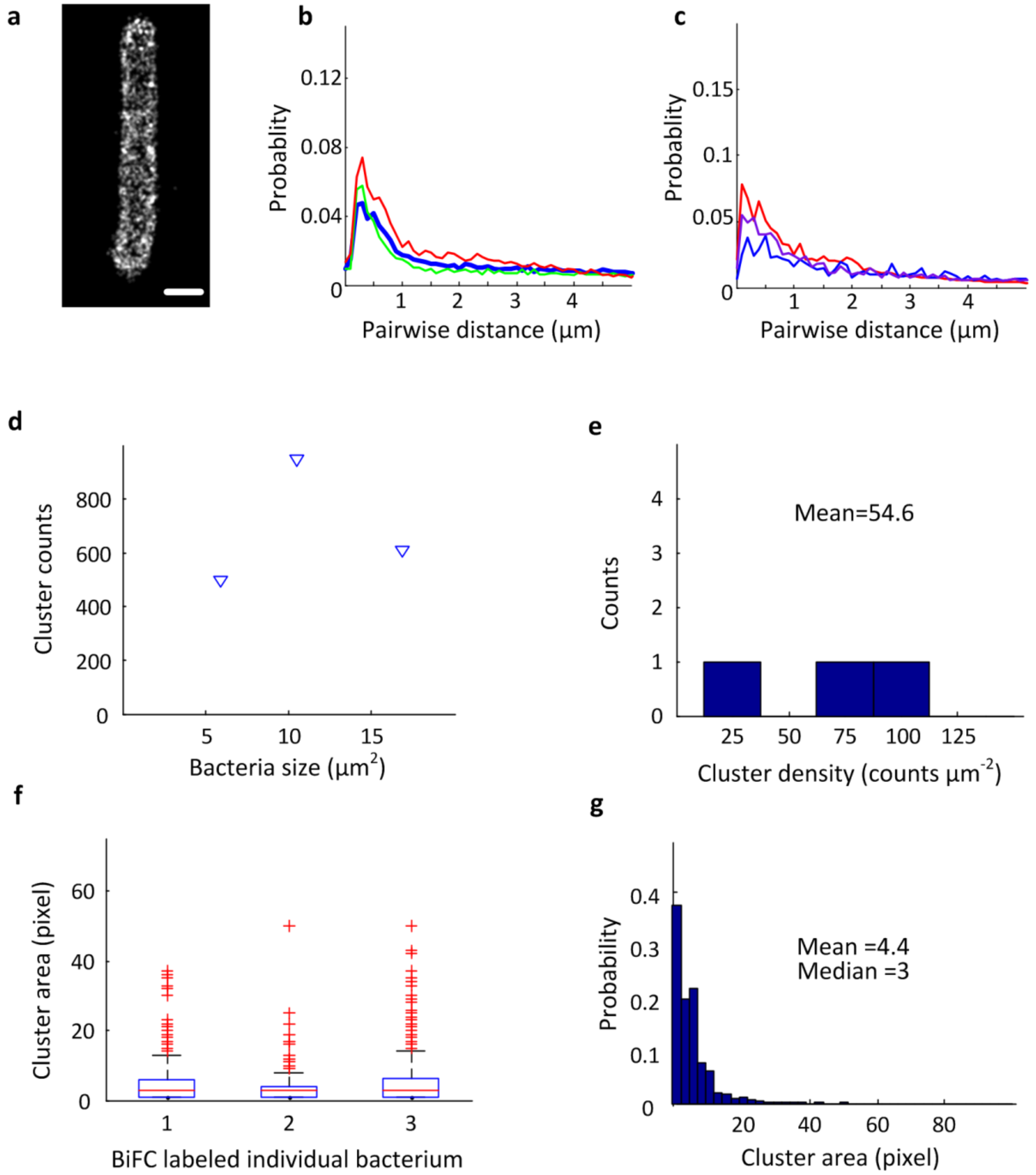

Supplementary Figure 8 | Cluster analysis of EFTu-MreB-PPI super-resolution images of elongated bacterial cells. (a) One bacterial cell demonstrated elongated shape; (b-g) Cluster analysis of the super-resolution images. (b) Pairwise distance distribution of all dots in the bacteria; (c) Pairwise distance distribution of all clusters in the bacteria; (d) Number of clusters in each bacterium as a function of the cell size; (e) Cluster density distribution of all 3 bacteria; (f) Median cluster area of each bacterium; (g) Cluster area distribution of all 3 bacteria. Scale bar 1 μm.

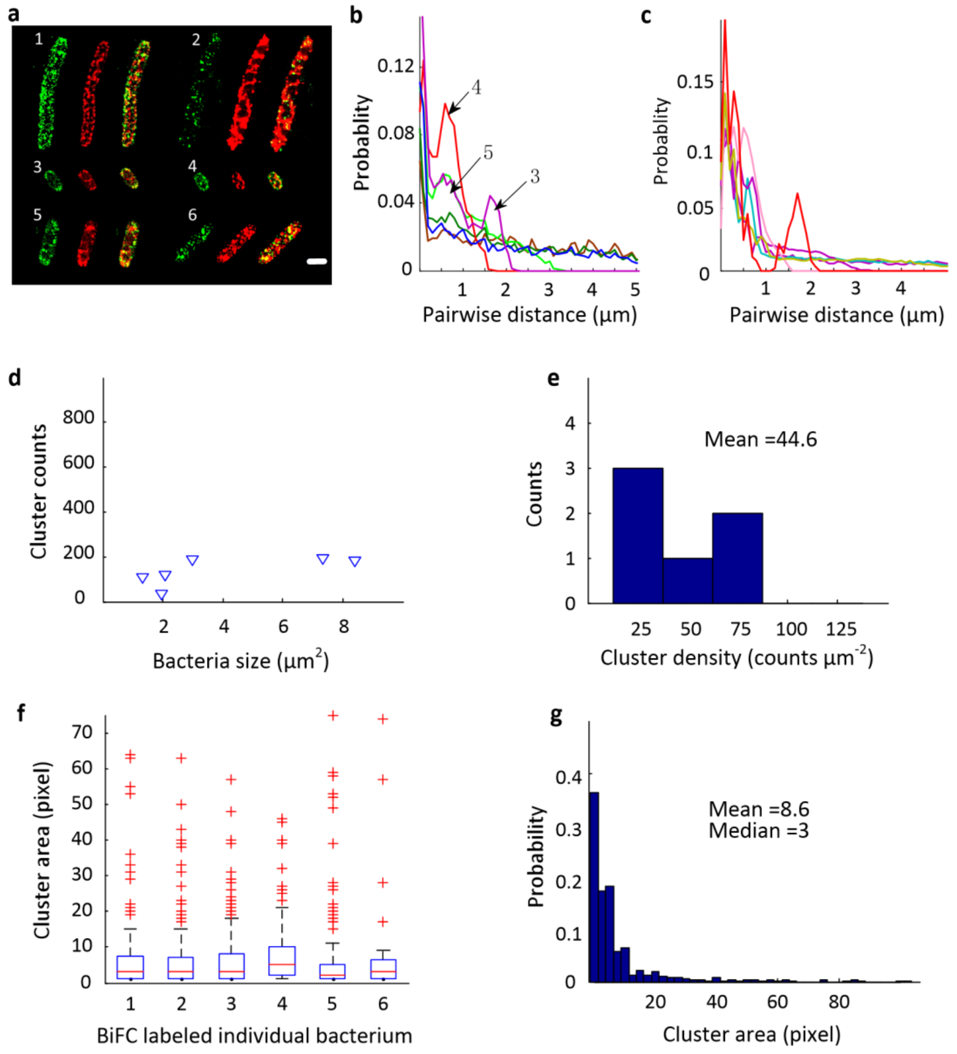

Supplementary Figure 9 | Cluster analysis of EFTu-MreB-PPI super-resolution images of the bacterial cells that also had EF-Tu labeled with BG-Alexa647 on the Snap-tag. (a) 6 bacterial cells demonstrated different patterns for EFTu-MreB-PPIs, including patch-like and polar localization. Green represents the BiFC signal, red represents the EF-Tu signal and yellow represents the overlap; (b-h) Cluster analysis of the super-resolution images. (b) Pairwise distance distribution of all dots in the bacteria. The peaks marked by "3, 4, and 5" suggest that EFTu-MreB-PPIs likely formed many clusters that were separated by about 700nm in bacteria #3, 4, and 5 in panel (a); (c) Pairwise distance distribution of all clusters in the bacteria; (d) Number of clusters in each bacterium as a function of the cell size; (e) Cluster density distribution of all 6 bacteria; (f) Median cluster area of each bacterium; (g) Cluster area distribution of all 6 bacteria. Scale bar 1 μm.

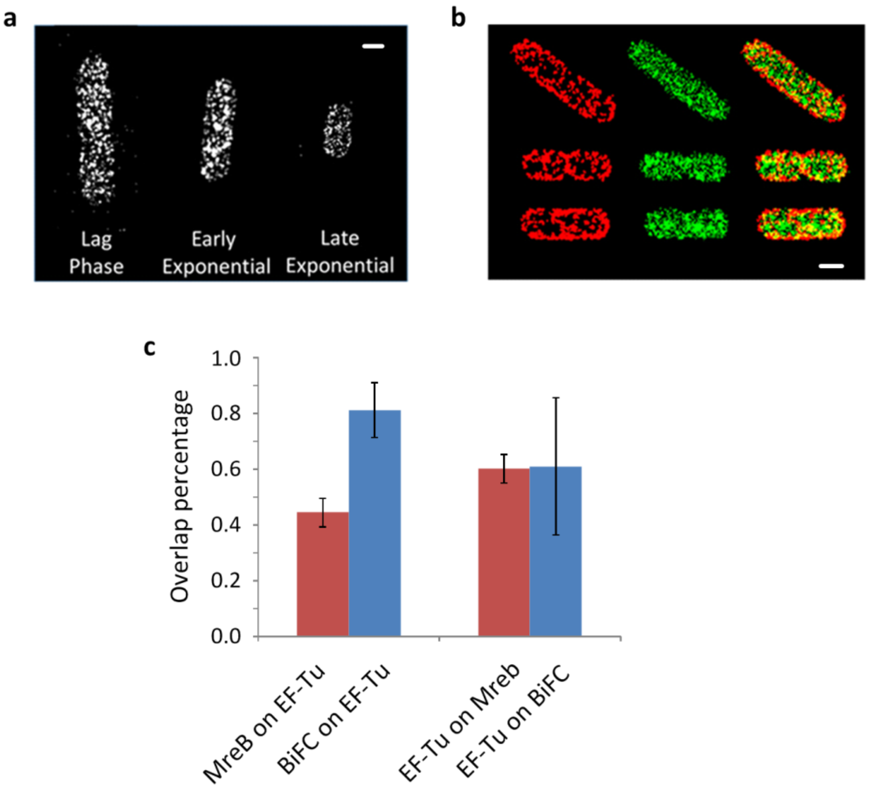

Supplementary Figure 10 | Colocalization analysis of MreB and EF-Tu. (a) PALM imaging of EF-Tu-mEos2 in 3 fixed bacterial cells at different growth phases; (b) Immuno-labeled MreB (red) and EF-Tu-mEos2 (green) in 3 fixed bacterial cells; (c) Pixel-overlapping analysis calculates, for instance "MreB on EF-Tu", the percentage of MreB red pixels that are also occupied by EF-Tu green out of the total number of red pixels. EFTu-MreB-PPI BiFC as a subpopulation of the total EF-Tu showed a significantly higher overlap percentage (the first blue bar) than that of "MreB on EF-Tu" (the first red bar). In contrast, "EF-Tu on MreB" and "EF-Tu on BiFC" showed very similar overlap percentage, likely as a convolution of specific EFTu-MreB interaction and random colocalization due to high EF-Tu density. Scale bar 1μm.

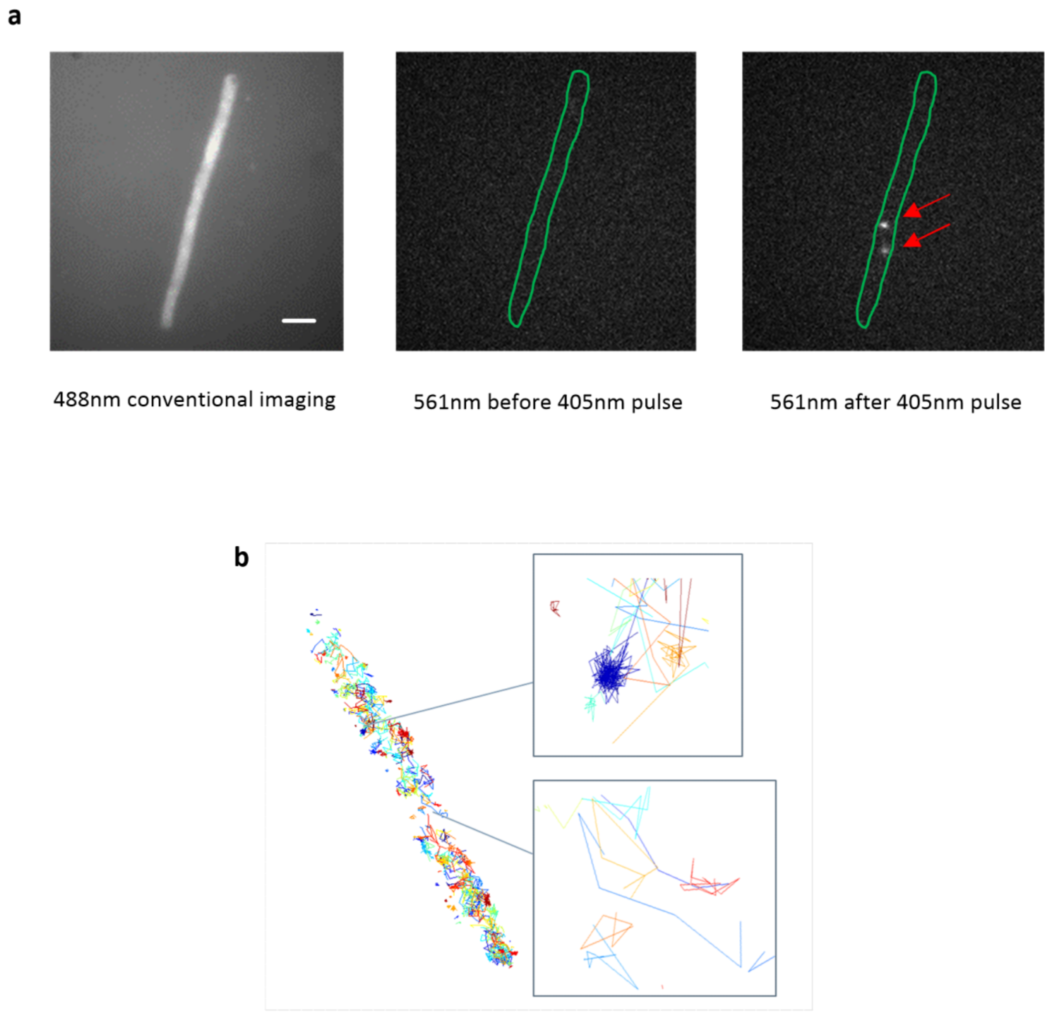

Supplementary Figure 11 | BiFC-PALM single molecule tracking of EFTu-MreB-PPIs in live *E. coli* cells. **(a)** 488nm illumination excited all reconstituted mEos3.2 in EFTu-MreB-PPIs. The excess fluorescent background made it impossible to see mobile single molecules (left panel). Photo-controlled convertibility allowed mEos3.2 molecules to be sparsely turned on and single molecules were able to be tracked (middle and right panels, also see Movie 2); **(b)** Repeated activation and tracking provided a motion trajectory map of EFTu-MreB-PPIs in live *E. coli* cells, which can be used to analyze localization-dependent dynamics of interacting protein pairs (see Fig. 3c). Scale bar 1 μm.

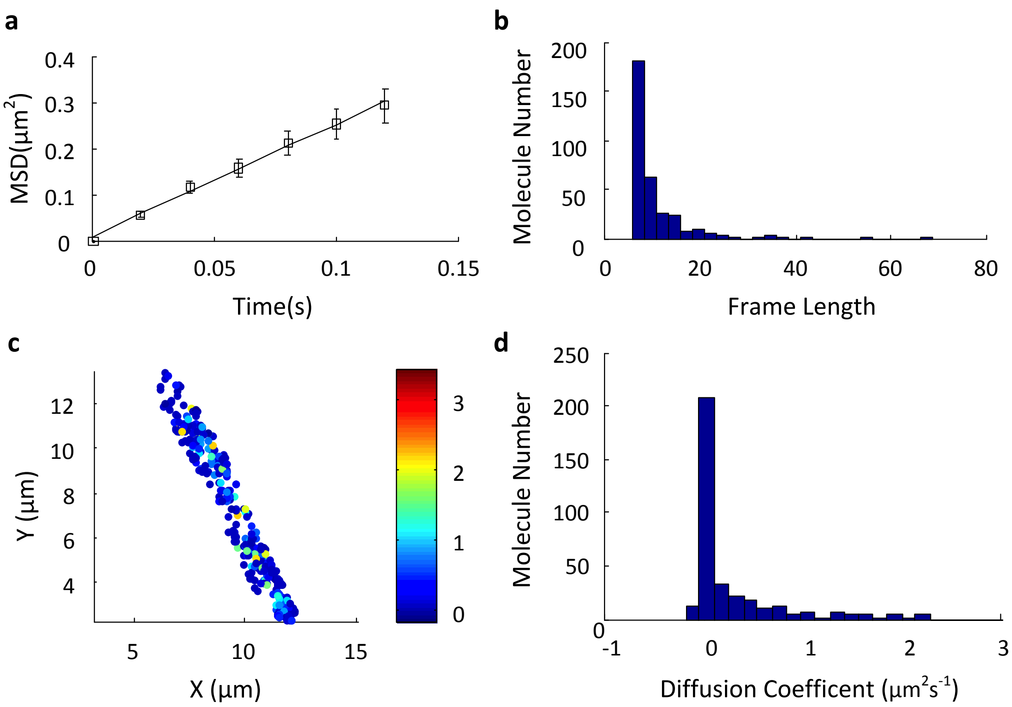

Supplementary Figure 12 | Quantification of single EFTu-MreB-PPI dynamics in live *E. coli* cells using BiFC-PALM single molecule tracking. **(a)** Mean squared displacements (MSD) as a function of time for the group of fast trajectories (red in Fig. 3b) provides the diffusion constant of the relative mobile group of EFTu-MreB-PPIs; **(b)** The histogram of the EFTu-MreB-PPIs that lasted longer than 6 frames (0.12s) under the experimental condition; **(c)** The diffusion coefficient map of EFTu-MreB-PPIs in a live bacteria cell; **(d)** The diffusion constant distribution for all trajectories.

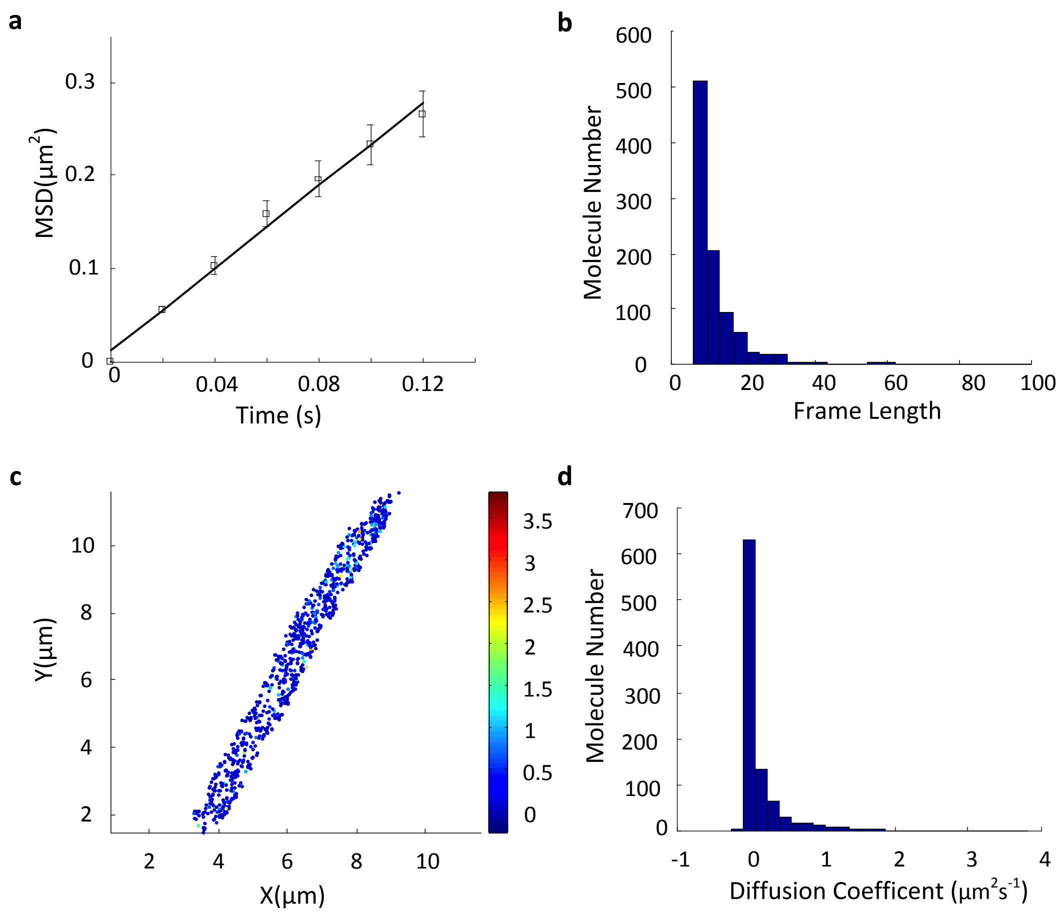

Supplementary Figure 13 | Quantification of single MreB dynamics in live *E. coli* cells using PALM single molecule tracking. **(a)** MSD as a function of time for the group of fast trajectories (red in Fig. 4b); **(b)** The histogram of MreB that lasted longer than 6 frames (0.12s); **(c)** The diffusion coefficient map of MreB in a live bacteria cell; **(d)** The diffusion constant histogram for all trajectories.

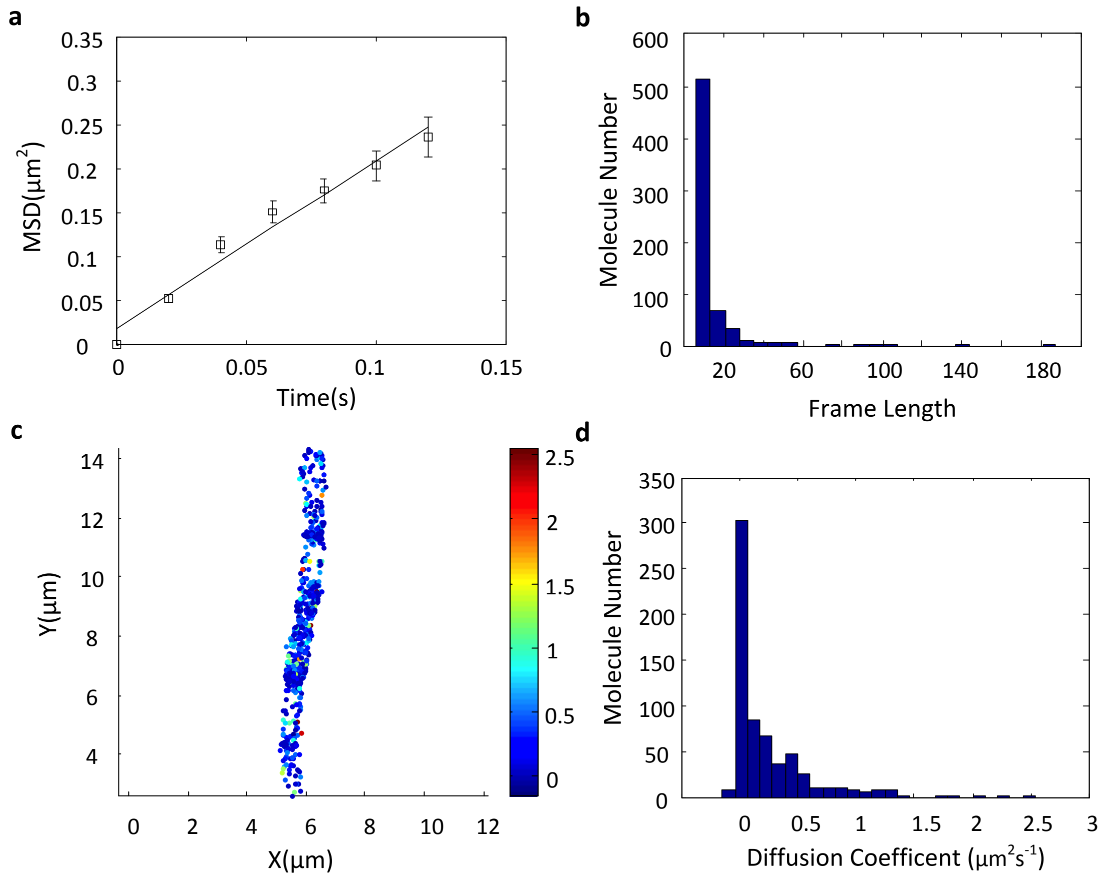

Supplementary Figure 14 | Quantification of single EF-Tu dynamics in live *E. coli* cells using PALM single molecule tracking. **(a)** MSD as a function of time for the group of fast trajectories (red in Fig.4e); **(b)** The histogram of MreB that lasted longer than 6 frames (0.12s); **(c)** The diffusion coefficient map of EF-Tu in a live bacteria cell; **(d)** The diffusion constant histogram for all trajectories.

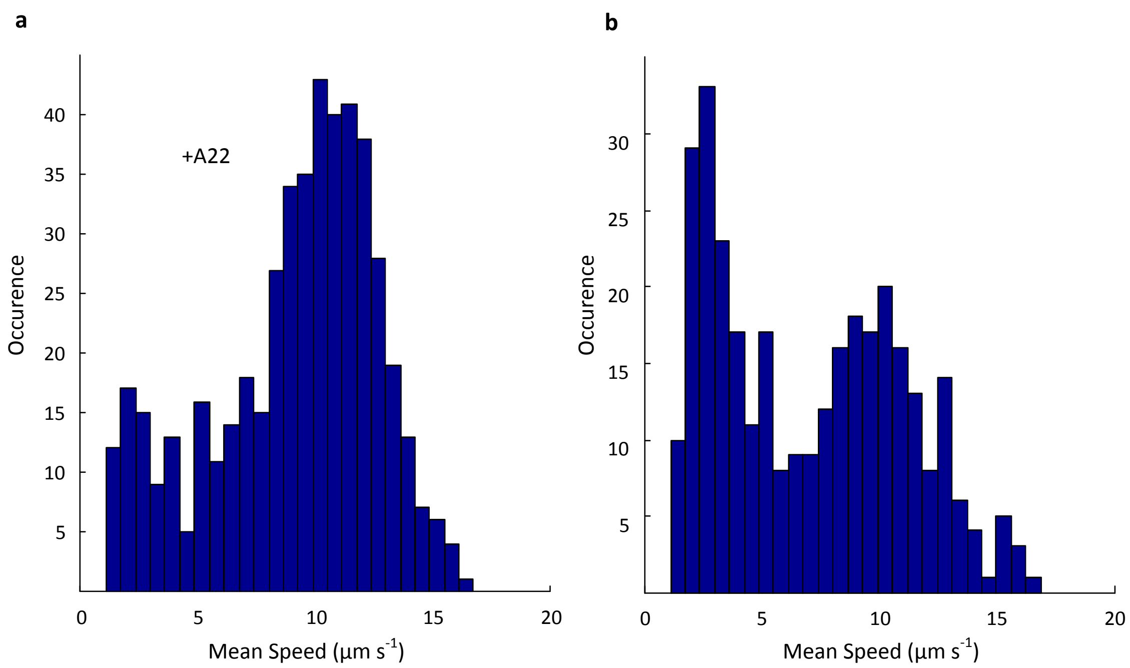

Supplementary Figure 15 | The motility populations were dependent on the polymerization state of MreB. (a) The MreB perturbing compound A22 decreased the fraction of slow mobility BiFC group and increased the fraction of fast mobility BiFC group, compared with that in the strain without addition of A22 (b).

a

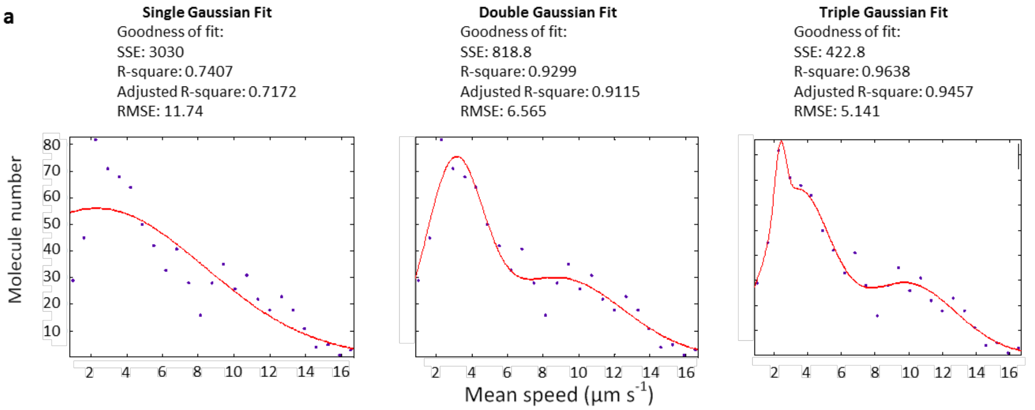

	Number of parameters in model	Remaining degrees of freedom	Sum of squares of residuals	Mean square
Double Guassian	6	19	818.8	43.09
Triple Guassian	9	16	422.8	26.43
Difference	3	3	396.00	132.00
F_value	5.00			

	Number of parameters in model	Remaining degrees of freedom	Sum of squares of residuals	Mean square
Single Guassian	3	22	3030	137.73
Double Guassian	6	19	818.8	43.09
Difference	3	3	2211.20	737.07
F_value	17.10			

b

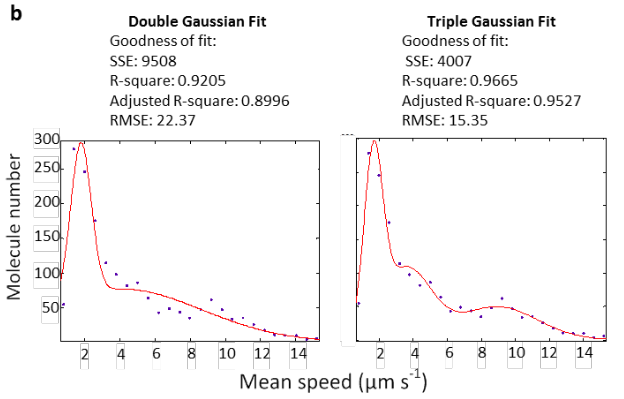

	Number of parameters in model	Remaining degrees of freedom	Sum of squares of residuals	Mean square
Double Guassian	6	19	9508	500.42
Triple Guassian	9	16	4007	250.44
Difference	3	3	5501.00	1833.67
F_value	7.32			

c

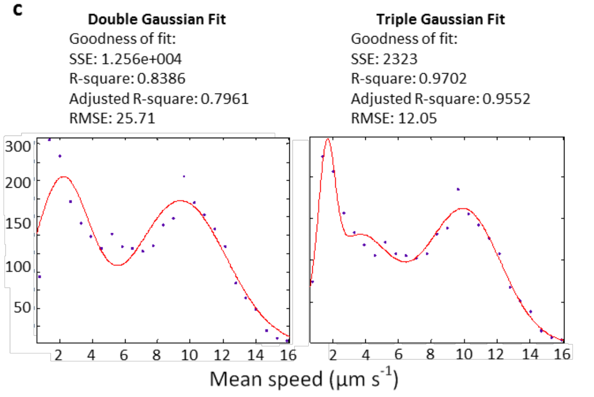

	Number of parameters in model	Remaining degrees of freedom	Sum of squares of residuals	Mean square
Double Guassian	6	19	12560	661.05
Triple Guassian	9	16	2323	145.19
Difference	3	3	10237.00	3412.33
F_value	23.50			

d

Triple Gaussian Fit	EFTu	MreB	EFTu-MreB
Peak#1	1.63	1.68	1.97
Peak#2	3.53	3.6	3.37
Peak#3	9.96	8.94	9.87

Supplementary Figure 16 | Evaluation of the fitting significance by F tests. (a) Evaluation of the fitting significance by single, double and triple Gaussian functions for the distribution in Fig.3b using F test; (b-c) Evaluation of the fitting significance by double and triple Gaussian functions for the distribution in Figs.4b&e using F tests; (d) Peak values of triple Gaussian fitting to the data of EF-Tu, MreB, and EFTu-MreB, respectively.

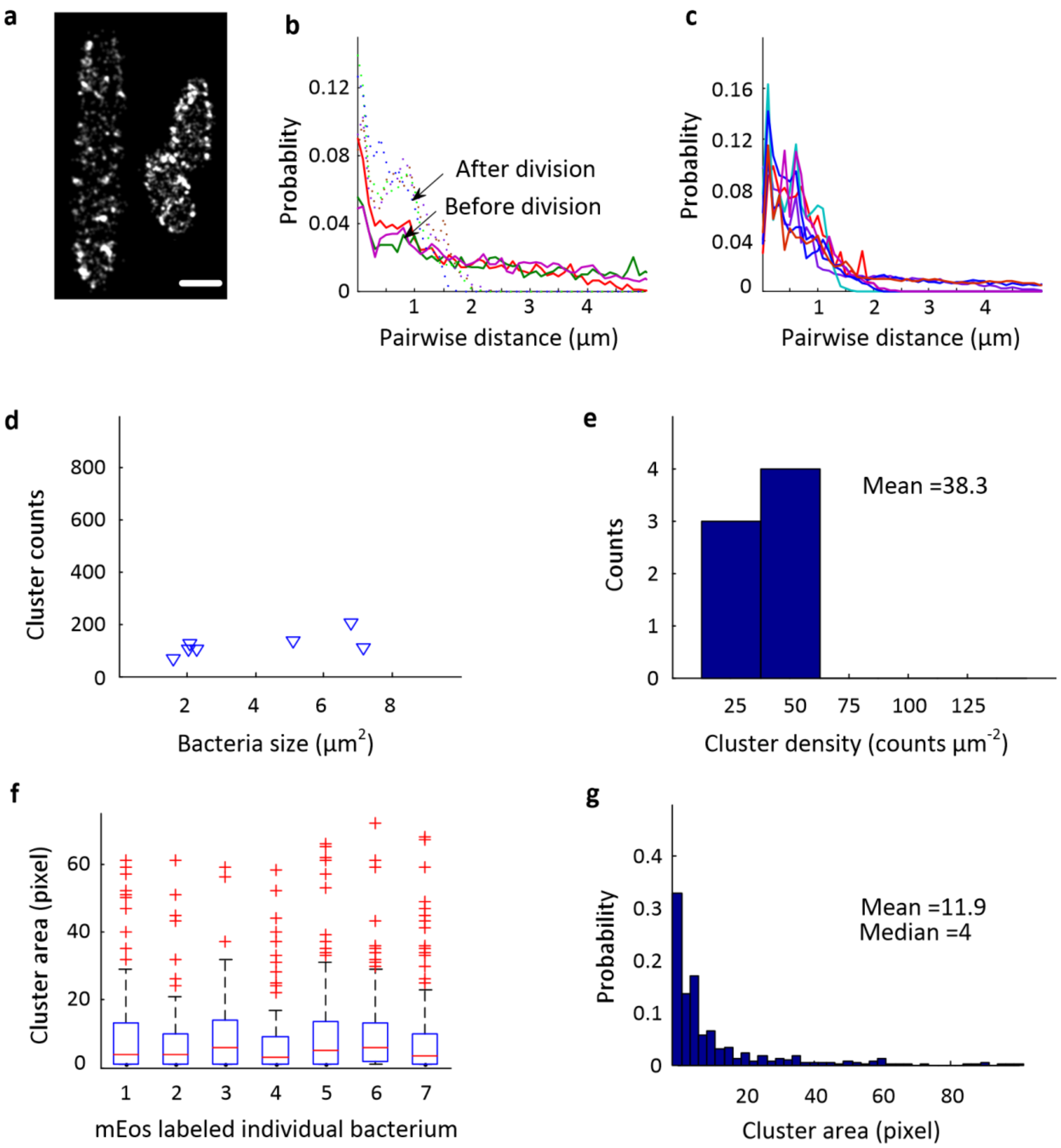

Supplementary Figure 17 | Super-resolution imaging and cluster analysis of MreB. (a) PALM imaging of MreBmEos3.2SW at different growth phases of *E. coli* cells, Scale bar 1 μm.; (b-g) Cluster analysis of 7 such bacterial cells. (b) Pairwise distance distribution suggests that MreB was organized differentially at different growth phases; (c) Pairwise distance distribution of all clusters in the bacteria; (d) Number of clusters in each bacterium as a function of the cell size; (e) Cluster density distribution of all 7 bacteria; (f) Median cluster area of each bacterium; (g) Cluster area distribution of all 7 bacteria.

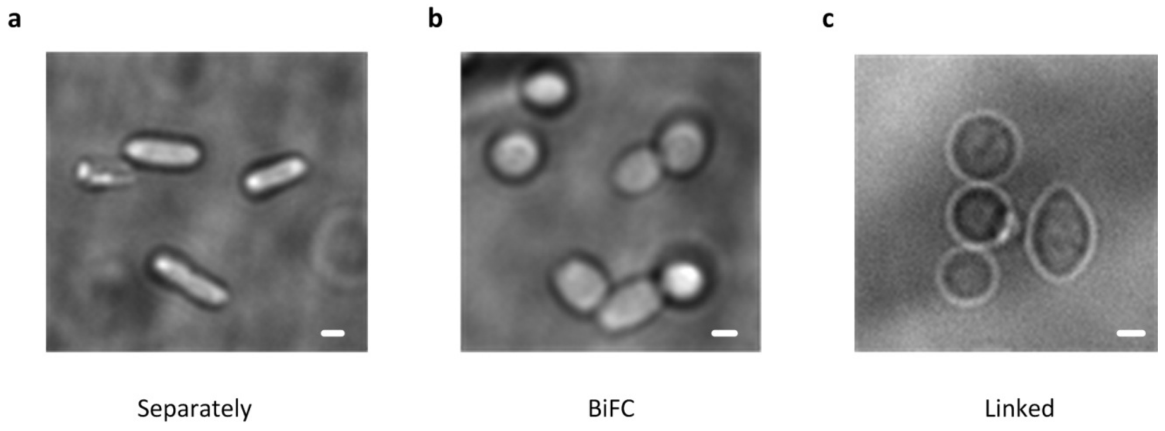

Supplementary Figure 18 | Bright field images showing different phenotypes of different constructs. (a) When both MreB and mEosN-EFTu were over-expressed, the bacterial cells looked normal; (b) Over-expression of BiFC EFTu-MreB induced bacterial cells into a ball-shape; (c) When MreB and EF-Tu were tethered with a linker peptide and over-expressed, the bacterial cells also tended to grow into a ball-shape. Scale bar 1 μm.

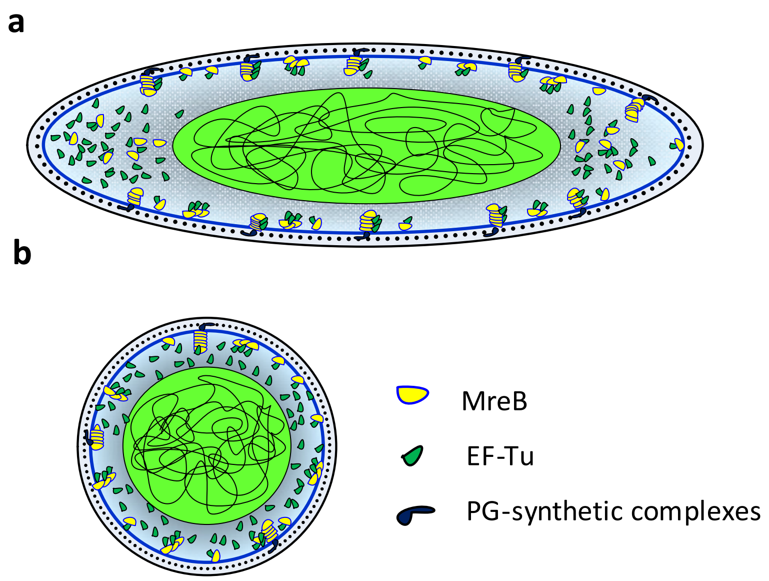

Supplementary Figure 19 | Models for the roles of MreB, EF-Tu, and MreB-EF-Tu pairs in cell wall synthesis and cell shape maintenance. (a) MreB molecules polymerize into filaments and move underneath the inner membrane to guide cell-wall insertion. EF-Tu molecules also likely polymerize and interact with MreB filaments on the inner membrane; (b) Binding of EF-Tu to MreB might prevent MreB from polymerizing into long filaments, which causes the cell rounding by missing directed cell-wall synthesis as well as impaired mechanical rigidity of the cell.

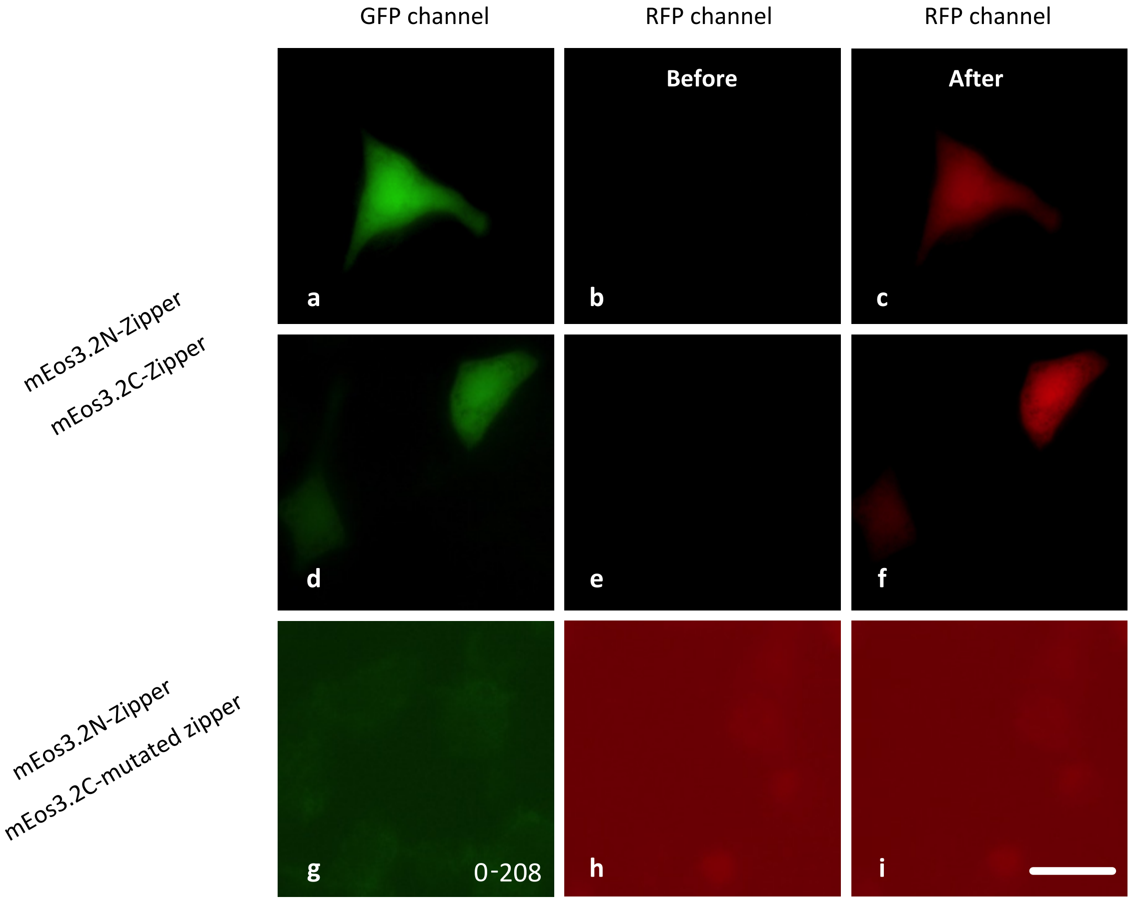

Supplementary Figure 20 | mEos3.2- BiFC analysis of Zipper interactions in living HeLa cells. HeLa cells co-transfected with mEos3.2N-Zipper and mEos3.2C-Zipper or mEos3.2C-mutated zipper plasmids imaged under GFP channel (a, d, g) and RFP channel (b, e, h). In order to test the photo-conversion capability of mEos3.2-BiFC-Zipper complexes, the cells were illuminated with UV light (Excitation filter 387/11, Semrock) for 10 seconds and imaged with RFP channel (c, f, i) again. Before: before UV light activation (b, e, h). After: after UV light activation (c, f, i). All images were taken with 500ms exposure time and 0-208 indicates the gray level range of (g, h,i). Scale bar 10 μm.

Supplementary Table 1: Photophysical properties of complemented mEos3.2 compared with native mEos3.2**.**

**Brightness**	Green form	Red form
Native mEos3.2	53	18
Complemented mEos3.2	62.7	18.3
**Quantum Yields**	Green form	Red form
Native mEos3.2	0.84	0.55
Complemented mEos3.2	0.81	0.54
**Excitation/Emission**	Green form	Red form
Native mEos3.2	507/516	572/580
Complemented mEos3.2	505/516	570/580
**Photoconversion efficiency**	k_{on}（s^{-1}）	
Native mEos3.2	0.01	
Complemented mEos3.2	0.0104	

Supplementary Table 2: Estimation of the copy numbers

	EF-Tu	MreB	EFTu-MreB-PPI
Copy #	~90,000	~30,000	(MreB-EF-Tu) / (EF-Tu) ≃ 16%
Low mobility% (#) (v ≈ 1.7 μm s^{-1})	10.9% (9810)	34.0% (10200)	4.3% (620)
Intermediate mobility % (#) (v ≈ 3.5 μm s^{-1})	38.3% (34470)	37.3% (11190)	57.1% (8220)
High mobility % (#) (v ≈ 10 μm s^{-1})	50.9% (45810)	28.7% (8610)	38.6% (5560)

Supplementary Table 2: Estimation of the copy numbers for different mobility populations of EF-Tu, MreB, and EFTu-MreB-PPIs. Intensity analysis: in single molecule localization based super-resolution imaging methods such as STORM and PALM, the pixel intensity in the reconstructed super-resolution image is proportional to the number of photo-activation/photo-conversion events. Therefore, one may count how many molecules in any diffraction limited area (Puchner et al., Counting molecules in single organelles with superresolution microscopy allows tracking of the endosome maturation trajectory, PNAS 2013). The complication here is that mEos3.2 was only photo-converted once before photo-bleaching, while Alexa647 can be photo-switched multiple times. We carried out the following estimation for intensity analysis. Firstly, we did pixel-overlapping analysis as in Supplementary Figure 10c to quantify the colocalized area. Secondly, we compared the total intensity of colocalized EFTu-Snap area (red intensity of the yellow area in Fig.3c) with that of the total EFTu-Snap area to estimate the fraction of EF-Tu that interacted with MreB.

Supplementary Table 3: Primer list.

Purpose	Name	Sequence(5' to 3')
**7 Splits**	NZEos5	CATGCCATGGGCAGTGCGATTAAGCCAGACATGAAGATCAAAC
	NZEos3	CGGAATTCTTACTGGGCCAGTTCCTTTTTCAGGGC
	CZEos5	GGAAGATCTCGAACAACTGGAGAAAAAGCTGCAGGCG
	CZEos3	CCGCTCGAGTTATCGTCTGGCATTGTCAGGCAATCCAG
	NZ-3	TTACTGGGCCAGTTCCTTTTTCAGCGCCTGCAGTTCCCATTTCAGCTGCGCCAG CTCTTTCTTGTTCGCCTGTAATTCTTTCTTCAGC
	CZ-5	GAACAACTGGAGAAAAAGCTGCAGGCGCTGGAAAAGAAACTGGCGCAGCTG GAATGGAAGAACCAGG
	N34F3	TTTCTTGTTCGCCTGTAATTCTTTCTTCAGCGCACCGCTGCCGCTACCGCCAAAA GGCTTGCCTGTACCATCTCCGTC
	C34F5	CGCAGCTGGAATGGAAGAACCAGGCGCTGGAAAAGAAACTGGCGCAGGGCG GTAGCGGCGAGGGAAAACAGAGTATGGATCTTGAAGTCAAAG
	N96E3	TTTCTTGTTCGCCTGTAATTCTTTCTTCAGCGCACCGCTGCCGCTACCGCCTTCG AAAGTCAAGCTTCGTTCCCACG
	C96E5	CGCAGCTGGAATGGAAGAACCAGGCGCTGGAAAAGAAACTGGCGCAGGGCG GTAGCGGCGACGGGGGCATTTGCATTGCCAG
	N138K3	TTTCTTGTTCGCCTGTAATTCTTTCTTCAGCGCACCGCTGCCGCTACCGCCTTTC AGCGTCTTCTTCTGCATAACTGGACC
	C138K5	CGCAGCTGGAATGGAAGAACCAGGCGCTGGAAAAGAAACTGGCGCAGGGCG GTAGCGGCTGGGAGCCCTCCACTGAGAAAATGTATG
	N148V3	TTTCTTGTTCGCCTGTAATTCTTTCTTCAGCGCACCGCTGCCGCTACCGCCCACA TACATTTTCTCAGTGGAGGGCTCCC
	C148V5	CGCAGCTGGAATGGAAGAACCAGGCGCTGGAAAAGAAACTGGCGCAGGGCG GTAGCGGCCGTGATGGAGTGCTGACGGGTGATATTC
	N150D3	TTTCTTGTTCGCCTGTAATTCTTTCTTCAGCGCACCGCTGCCGCTACCGCCATCA CGCACATACATTTTCTCAGTGGAGGG
	C150D5	CGCAGCTGGAATGGAAGAACCAGGCGCTGGAAAAGAAACTGGCGCAGGGCG GTAGCGGCGGAGTGCTGACGGGTGATATTCATATGGC
	N160A3	TTTCTTGTTCGCCTGTAATTCTTTCTTCAGCGCACCGCTGCCGCTACCGCCAGCC ATATGAATATCACCCGTCAGCACTC
	C160A5	CGCAGCTGGAATGGAAGAACCAGGCGCTGGAAAAGAAACTGGCGCAGGGCG GTAGCGGCTTGTTGCTTGAAGGAAATGCCCATTACC
	N164E3	TTTCTTGTTCGCCTGTAATTCTTTCTTCAGCGCACCGCTGCCGCTACCGCCTTCA AGCAACAAAGCCATCTCAATATCACCC
	C164E5	CGCAGCTGGAATGGAAGAACCAGGCGCTGGAAAAGAAACTGGCGCAGGGCG GTAGCGGCGGAAATGCCCATTACCGATGTGACTTCAG
**BiFC Specificity**	Neg1-5'''	CGCAGCTGAAATGGAAGAACCAGGCGCTGAAAAAGAAACTGGCGCAGGGCG GTAGCGGCGGAAATGCCCATTACCGATGTGAC
	Neg1-5''	GAACAACTGGAGAAAAAGCTGCAGGCGCTGGAAAAGGAACTGGCGCAGCTG AAATGGAAGAACC
	Neg1-5'	GAACAACTGGAGAAAAAGCTGCAGG
	Neg2-5'''	CGCAGCTGAAATGGAAGAACCAGGCGCTGGAAAAGAAACTGGCGCAGGGCG GTAGCGGCGGAAATGCCCATTACCGATGTGAC

	Neg2-5''	GAACAACTGGAGAAAGAACTGCAGGCGCTGGAAAAGGAACTGGCGCAGCTGAAATGGAAGAACC
	Neg1-5'	GAACAACTGGAGAAAGAACTGCAGG
	Neg3	TTATCGTCTGGCATTGTCAGGCAATC
**MreB-mEosC**	MreB5	CTAGCTAGCATGTTGAAAAAATTTCGTGGCATGTTTTCC
	mEosC3	AACTGCAGTTATCGTCTGGCATTGTCAGGCAATC
**mEosN-EF-Tu**	mEosN5	CGGGATCCG ATGAGTGCGATTAAGCCAGACATGAAGAT
	mEosN3	GGAAAACATGCCACGAAATTTTTTCAACATACCGCTGCCGCTACCGCCTTCAAGCAACAAAGCCATCTCTACATCACC
	EF-Tu5	GGCGGTAGCGGCAGCGGTATGTTGAAAAAATTTCGTGGCATGTTTTCC
	EF-Tu3	AACTGCAG TTAGCTCAGAACTTTTGCTACAACGCCC
**MreB-mEosN**	MreB5	CTAGCTAGCTAGATGTTGAAAAAATTTCGTGGCATGTTTTCC
	mEosN5	GCGAAGAGGGCGGTAGCGGCATGAGTGCGATTAAGCCAGACATGAAG
	MreB3	CTCATGCCGCTACCGCCCTCTTCGCTGAACAGGTCGCCG
	mEosN3	CCGCTCGAGTTATTCAAGCAACAAAGCCATCTCAATATCACC
**EFTu-mEosC**	EFTu5	CGGGATCCGATGTCTAAAGAAAAGTTTGAACGTACAAAACCG
	mEosC5	TTCTGAGCGGCGGTAGCGGCGGAAATGCCCATTACCGATGTGACTTC
	EFTu3	CATTTCCGCCGCTACCGCCGCTCAGAACTTTTGCTACAACG
	mEosC3	AACTGCAGTTATCGTCTGGCATTGTCAGGCAATC
**mreC-mEos3.2C**	Cm1	CTAGCTAGCATGAAGCCAATTTTTAGCCGTGGCCC
	Cm2	TCGGTAATGGGCATTTCCGCCGCTACCGCCTTGCCCTCCCGGCGCAC
	Cm3	CGTGCGCCGGGAGGGCAAGGCGGTAGCGGCGGAAATGCCCATTACCGATGTGACTT
	Cm4	CCGCTCGAGTTATCGTCTGGCATTGTCAGGCAATCC
**mreD-mEos3.2C**	Dm1	CTAGCTAGCATGGCGAGCTATCGTAGCCAGGGAC
	Dm2	TCGGTAATGGGCATTTCCGCCGCTACCGCCTTGCACTGCAAACTGCTGACGGACTT
	Dm3	CAGCAGTTTGCAGTGCAAGGCGGTAGCGGCGGAAATGCCCATTACCGATGTGACTT
	Dm4	CCGCTCGAGTTATCGTCTGGCATTGTCAGGCAATC
**EF-Tu-mEos2**	EFmEos5	GGCGGCCGTACCGTTGGCGCGGGCGTTGTAGCAAAAGTTCTGAGCATGAGTGCGATTAAGCCAGACATGAAG
	EFmEos3	AAATGATGCCCTTTTAGTGCGCATTGCGTCAAATGTTATCGGCAAGCCATGGTCCATATGAATATCCTCCTTAG
**MreB Snap**	MreBsnap5	CTGGAAATGATCGACATGCACGGCGGCGACCTGTTCAGCGAAGAGATGGACAAAGATTGCGAAATGAAACG
	MreBsnap3	TCGGATGCAGGCAGGGGAAGTGTCTGTTTACCCTGCCTGGTCTGAGTCCATATGAATATCCTCCTTAGTTC
**MreB-mEosC strain**	MC1	CGCTGGAAATGATCGACATGCACGGCGGCGACCTGTTCAGCGAAGAGGGCGGTAGCGGCGGAAATGC
	MC2	CTCGTATCAGACCAGGCAGGGTAAACAGACACTTCCCCTGCCTGCATCCGAGCCATGGTCCATATGAATATCCTCCTTAG
**pbad-mEosN-tufB-Snap**	snap1	AACTGCAGCTATGAGTGCGATTAAGCCAGACATGAAGAT
	snap2	GTCCATGCCGCTACCGCCGCTCAGAACTTTTGCTACAACGCCC
	snap3	CTGAGCGGCGGTAGCGGCATGGACAAAGATTGCGAAATGAAACG

	snap4	GGAATTC TTATCCCAGACCCGGTTTACCCAGAC
**BiFC-Venus**	VN_Z_5’	CATGCCATGGGCGTGAGCAAGGGCGAGGAGCTGT
	VN_Z_3’	GGCGGTGATATAGACGTTGTGGCTG
	VN_Z_3’’’	TTTCTTGTTCGCCTGTAATTCTTTCTTCAGCGCACCGCTGCCGCTACCGCCGGC GGTGATATAGACGTTGTGGCTG
	VN_Z_3”	CTGGGCCAGTTCCTTTTTCAGCGCCTGCAGTTCCCATTTCAGCTGCGCCAGCTC TTTCTTGTTCGCCTGTAATTCTTTC
	VN_Z_3’	CGGAATTCTTACTGGGCCAGTTCCTTTTTCAGG
	VC_Z_5’’’	CGCAGCTGGAATGGAAGAACCAGGCGCTGGAAAAGAAACTGGCGCAGGGCG GTAGCGGCGACAAGCAGAAGAACGGCATCAAGG
	VC_Z_5”	GAACAACTGGAGAAAAAGCTGCAGGCGCTGGAAAAGAAACTGGCGCAGCTG GAATGGAAGAACC
	VC_Z_5’	GGAAGATCTCGAACAACTGGAGAAAAAGCTGCAGG
	VC_Z_3’	CCGCTCGAGTTACTTGTACAGCTCGTCCATGCCGAGAG
**BiFC-Dronpa**	DN_Z_5’	CATGCCATGGGCGTGAGTGTGATTAAACCAGACATGAAG
	DN_Z_3’’’	TTTCTTGTTCGCCTGTAATTCTTTCTTCAGCGCACCGCTGCCGCTACCGCCTTCA AGCGACAGAGCCATGTTAACATCACC
	DN_Z_3”	CTGGGCCAGTTCCTTTTTCAGCGCCTGCAGTTCCCATTTCAGCTGCGCCAGCTC TTTCTTGTTCGCCTGTAATTCTTTC
	DN_Z_3’	CGGAATTCTTACTGGGCCAGTTCCTTTTTCAGG
	DC_Z_3’	CCGCTCGAGCTTGGCCTGCCTCGGCAGCTC
	DC_Z_5’’’	CGCAGCTGGAATGGAAGAACCAGGCGCTGGAAAAGAAACTGGCGCAGGGCG GTAGCGGCGGAGGTGGCCATTACCGATGTGACT
	DC_Z_5”	GAACAACTGGAGAAAAAGCTGCAGGCGCTGGAAAAGAAACTGGCGCAGCTG GAATGGAAGAACC
	DC_Z_5’	GGAAGATCTCGAACAACTGGAGAAAAAGCTGCAGG
**mEosN-EFTu-ΔN ΔC**	MtdNdC_1	CGCGGATCCGATGAGTGCGATTAAGCCAGACATGAAGAT
	MtdNdC_2	ACCGCTGCCGCTACCGCCTTCAAGCAACAAAGCCATCTCTACATCACC
	MtdNdC_3	GGCGGTAGCGGCAGCGGTCAGATCGATAACGCGCCGG
	MtdNdC_4	AAAACTGCAGTTACGCGATCGGGTGGATCAGGGT
